# Impact of genetic patterns on sorafenib efficacy in patients with FLT3-ITD acute myeloid leukemia undergoing allogeneic hematopoietic stem cell transplantation: a multi-center, cohort study

**DOI:** 10.1038/s41392-023-01614-1

**Published:** 2023-09-14

**Authors:** Ruoyang Shao, Yu Zhang, Jinping He, Fen Huang, Zhiping Fan, Kaibo Yang, Yajing Xu, Na Xu, Yi Luo, Lan Deng, Xi Zhang, Jia Chen, Mingzhe Han, Xudong Li, Sijian Yu, Hui Liu, Xinquan Liang, Xiaodan Luo, Pengcheng Shi, Zhixiang Wang, Ling Jiang, Xuan Zhou, Ren Lin, Yan Chen, Sanfang Tu, Jing Sun, Yu Wang, Qifa Liu, Li Xuan

**Affiliations:** 1 Department of Hematology, Nanfang Hospital, Southern Medical University, Guangzhou, 510515 China; 2 Clinical Medical Research Center of Hematology Diseases of Guangdong Province, Guangzhou, 510515 China; 3 Department of Hematology, Xiangya Hospital, Central South University, Changsha, 410008 China; 4 Bone Marrow Transplantation Center, the First Affiliated Hospital, Zhejiang University School of Medicine, Hangzhou, 310009 China; 5 Department of Hematology, Zhujiang Hospital, Southern Medical University, Guangzhou, 510280 China; 6 Department of Hematology, Shanghai Ninth People’s Hospital, Shanghai Jiao Tong University School of Medicine, Shanghai, 200125 China; 7 Department of Hematology, Xinqiao Hospital, Third Military Medical University, Chongqing, 400037 China; 8 The First Affiliated Hospital of Soochow University, Suzhou, 215006 China; 9 Hematopoietic Stem Cell Transplantation Center, Institute of Hematology and Blood Diseases Hospital, Peking Union Medical College and Chinese Academy of Medical Sciences, Tianjin, 300020 China; 10 Department of Hematology, the Third Affiliated Hospital of Sun Yat-Sen University, Guangzhou, 510630 China; 11 Department of Hematology, the First People’s Hospital of Chenzhou, Chenzhou, 423099 China; 12 Department of Hematology, the First Affiliated Hospital of Guangzhou Medical University, Guangzhou, 510120 China; 13 Department of Hematology, the Fifth Affiliated Hospital of Guangzhou Medical University, Guangzhou, 510799 China; 14 Department of Hematology, Peking University People’s Hospital, Beijing, 100044 China; 15 Guangdong Provincial Key Laboratory of Digital Medicine and Biomechanics, National Key Discipline of Human Anatomy, School of Basic Medical Sciences, Southern Medical University, Guangzhou, 510515 China

**Keywords:** Haematological cancer, Haematological cancer

## Abstract

Sorafenib therapy improves overall survival (OS) in patients with FLT3 internal tandem duplication (ITD) acute myeloid leukemia (AML) undergoing allogeneic hematopoietic stem cell transplantation. We explored the efficacy of sorafenib therapy in this population with different concomitant genetic patterns. In this multi-center, cohort study, we enrolled patients with FLT3-ITD AML undergoing allogenic hematopoietic cell transplantation. Patients with sorafenib maintenance post-transplantation for at least four weeks were allocated to the sorafenib group, and otherwise to the control group. Endpoints were OS, disease-free survival, and relapse for the whole cohort and OS for genetic pattern subgroups. Among 613 patients enrolled, 275 were in the sorafenib and 338 the control group. Median follow-up was 36.5 (interquartile range (IQR), 25.2–44.7) months post-transplantation. The 3-year OS post-transplantation was 79.6% (95% confidential interval (CI) 74.8%–84.6%) and 65.2% (95% CI 60.3%–70.6%) (Hazard ratio (HR) 0.50, 95% CI 0.37–0.69; *P* < 0.0001) in both groups. Sorafenib maintenance post-transplantation improved OS in the favorable (HR 0.33, 95% CI 0.14–0.77; *P* = 0.011) and adverse (HR 0.56, 95% CI 0.33–0.93; *P* = 0.026) ELN 2017 risk subgroups. Patients with mutated NPM1, DNMT3A, co-occurring NPM1/DNMT3A, “activated signaling” and “DNA methylation” genes benefited in OS from sorafenib maintenance, while those carrying CEBPA, “tumor suppressors” and “myeloid transcription factors” genes did not. Patients with FLT3-ITD^high^ and FLT3-ITD^low^ AML both benefited in OS from sorafenib maintenance. Our results identify the response of genetic patterns to sorafenib maintenance, providing new viewpoints for the optimal use of sorafenib in FLT3-ITD AML in the transplantation setting.

## Introduction

FMS-like tyrosine kinase 3 (FLT3), a transmembrane ligand-activated receptor tyrosine kinase, is usually expressed by hematopoietic stem cells, early myeloid progenitor cells, and early lymphoid progenitor cells.^1^ FLT3 plays a key role in the regulation of proliferation, maturation and apoptosis in especially the early stages of hematopoietic cells via multiple downstream signaling pathways including JAK-STAT, RAS/RAF/MEK/ERK, and PI3K/AKT.^2^ FLT3 is among the most frequently mutated genes in newly diagnosed acute myeloid leukemia (AML), including two main types of mutation, FLT3 juxta-membrane domain internal tandem duplication (ITD), and FLT3 tyrosine kinase domain (TKD) point mutation.^2^ An ITD in FLT3 molecule inhibits the negative regulatory function of the juxta-membrane region, resulting in constitutive activation of the FLT3 kinase and its downstream signaling cascades, and consequently improves survival and proliferation of AML cells.^2^ While the clinical impact of FLT3-TKD is still ambiguous, patients with FLT3-ITD positive AML face shorter remission duration and higher relapse compared to those with wild type FLT3.^3^ Therefore, allogeneic hematopoietic stem cell transplantation (allo-HSCT) is recommended by international consensus and guidelines for FLT3-ITD AML patients, but many patients still undergo relapse even after allo-HSCT.^4,5^

Recent years, several different FLT3 inhibitors including sorafenib have been widely applied in FLT3-ITD AML, incorporated with chemotherapy, as maintenance therapy, and as salvage therapy.^2,6–12^ Prospective and retrospective studies including those from our group demonstrated that administration of sorafenib in FLT3-ITD AML patients, especially after allo-HSCT, could reduce relapse and improve survival.^9,13–20^ A recent updated report of our phase III randomized controlled trial (RCT) showed that sorafenib maintenance post-transplantation had long-term benefits on relapse rate and overall survival (OS) in FLT3-ITD AML patients receiving allo-HSCT without significantly increasing adverse events or graft-versus-host disease (GVHD).^19^ Sorafenib is now recommended by the AML guideline of the National Comprehensive Cancer Network (NCCN) and ELN as post-transplantation maintenance therapy for FLT3-ITD AML patients.^21,22^

FLT3-ITD AML is a group of heterogenous disease. Despite the number, length, insertion site, allelic ratio (AR) of FLT3-ITD itself, concomitant genetic abnormalities also influence the nature of FLT3-ITD AML.^18,23,24^ For example, some studies showed that the co-existence of NPM1 or CEBPA mutation inferred superior prognosis, but TET2 or DNMT3A mutation predicted inferior outcome in FLT3-ITD AML.^6,25–27^ Regarding the response to FLT3 inhibitors, several different FLT3 inhibitors were reported to improve the outcomes of FLT3-ITD AML patients with mutated NPM1,^18,28,29^ while their effects on patients with other genetic patterns were less reported. A recent study by Smith et al. showed that relapsed/refractory FLT3-mutated patients carrying DNA methylation/hydroxymethylation mutations like DNMT3A might benefit from gilteritinib.^28^ Jahn and his colleagues found that FLT3-mutated patients carrying chromatin modifiers and spliceosome mutations might benefit from midostaurin.^29^ However, these results were obtained in patients undergoing chemotherapy instead of allo-HSCT.^18,28,29^ Also, due to differences in molecular structure, sorafenib might also have different performance on sensitivity compared with gilteritinib or midostaurin.^30^ To the best of our knowledge, there currently lack large-scale study exploring the impact of concomitant genetic patterns on the efficacy of sorafenib as post-transplantation maintenance therapy in FLT3-ITD AML patients undergoing allo-HSCT.

To comprehensively explore the impact of concomitant genetic patterns to sorafenib efficacy in FLT3-ITD AML patients undergoing allo-HSCT, we herein perform a multi-center, exploratory, cohort study in patients with FLT3-ITD AML undergoing allo-HSCT.

In this study, we identified genetic abnormality subgroups who benefitted or did not benefit from sorafenib maintenance post-transplantation, and further explored the possible molecular mechanisms of sorafenib resistance in patients carrying certain genetic abnormalities via bioinformatic analyses and preliminary experiments. Our results provide new insights for the optimal use of sorafenib in FLT3-ITD AML based on concomitant genetic patterns.

## Results

### Clinical and Treatment characteristics

**Fig. 1:**
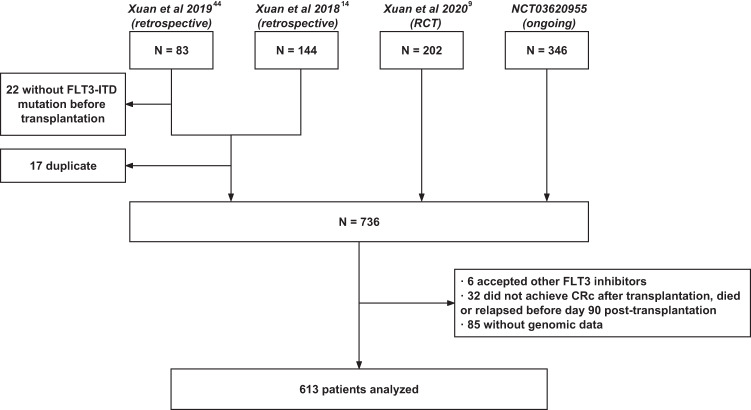
Diagram of the study. FLT3, FMS-like tyrosine kinase 3; FLT3-ITD, FMS-like tyrosine kinase 3 internal tandem duplication

A total of 613 patients with FLT3-ITD AML undergoing allo-HSCT were enrolled in this study between January 2012 and June 2020, including 441 patients from the prospective studies and 172 from the retrospective studies (Fig. 1). There were 294 females and 319 males, with a median age at 36 years (IQR, 26–45 years). For induction therapy, 562 patients received anthracyclines plus cytarabine and 51 patients received other regimens. At transplantation, 525 patients were in composite complete remission (CRc), 26 in partial remission (PR), and 62 in non-remission (NR). Two hundred and fifty-one patients underwent HLA-matched sibling donor (MSD), 42 HLA-matched unrelated donor (MUD) and 320 HLA-haploidentical donor (HID) transplants. ELN 2017 risk stratification was available in 505 patients, including 160 patients with favorable, 159 with intermediate and 186 with adverse risks, and was unavailable in 108 patients because of missing FLT3-ITD AR data. Based on sorafenib maintenance post-transplantation, 275 patients were allocated to the sorafenib group and 338 the control group. Except pre-transplantation sorafenib, prognostic factors such as sex, age, genetics, treatments pre-transplantation, disease status at transplant and transplant modality were balanced between the two groups (Table 1). The treatment and clinical characteristics were compared between patients from prospective and retrospective studies (Supplementary Table 1). Except cycles of chemotherapy pre-transplantation and ELN risk, characteristics were balanced between the prospective and retrospective cohorts (Supplementary Table 1). In the sorafenib group, sorafenib was initiated at a median of 45 days (IQR, 30–115 days) post-transplantation. The median time of sorafenib maintenance post-transplantation was 208 days (IQR, 144–292 days).

**Table 1:** Clinical and Treatment Characteristics

Characteristics	Sorafenib group (*n* = 275)	Control group (*n* = 338)	*P*
Sex, male/female (*n*%)	143 (53%)/132 (47%)	176 (52%)/162 (48%)	0.86
Age, median (IQR), years	36 (26–45)	37 (27–47)	0.24
WBC count at diagnosis, median (IQR)	53.1 (15.7–119.5)	52.9 (12.9–118.5)	0.96
Cycles of chemotherapy pre-transplant, median (IQR)	3 (3–4)	3 (3–4)	0.73
Initial induction regimens			0.25
Anthracyclines plus cytarabine	256 (93%)	306 (91%)	
Others	19 (7%)	32 (9%)	
Cytogenetics risk stratification (*n*%)			0.90
Favorable	20 (7%)	26 (8%)	
Intermediate	229 (83%)	279 (83%)	
Adverse	26 (9%)	33 (10%)	
2017 ELN risk stratification (*n*%)			0.90
Favorable	77 (28%)	83 (25%)	
Intermediate	84 (31%)	75 (22%)	
Adverse	83 (30%)	103 (30%)	
Unknown	31 (11%)	77 (23%)	
Disease status at transplant (*n*%)			0.79
CRc	238 (87%)	287 (85%)	
PR	10 (4%)	16 (5%)	
NR	27 (10%)	35 (10%)	
Sorafenib pre-transplant (*n*%)			0.028
Use	149 (54%)	153 (45%)	
No use	126 (46%)	185 (55%)	
Transplant modality (*n*%)			0.99
MSD	112 (41%)	139 (41%)	
MUD	22 (8%)	20 (6%)	
HID	141 (51%)	179 (53%)	

*WBC* white blood cell, *CRc* composite complete remission, *PR* partial remission, *NR* non-remission, *MSD* HLA-matched sibling donor, *MUD* HLA-matched unrelated donor, *HID* HLA-haploidentical donor

### Genetic landscape

**Fig. 2:**
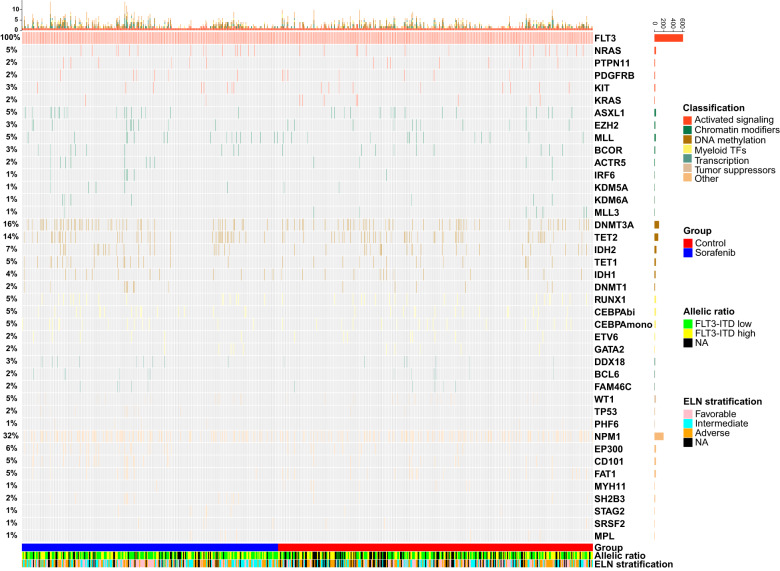
Genetic patterns of the whole population (mutation frequency ≥ 1%)

Of the 613 patients enrolled, Giemsa and reverse banding result was available in 577 patients, while fluorescence in-situ hybridization (FISH) data was available in all the patients. A total of 448 patients had a normal cytogenetics, and 165 an aberrant cytogenetics, including 46 favorable, 508 intermediate, and 59 adverse cytogenetics. Cytogenetic abnormality patterns were shown in Supplementary Fig. S1. For gene mutations, a total of 1285 concomitant mutations were detected. Four hundred and ten patients (67%) harbored at least one concomitant mutation, along with 191 (69%) and 219 (65%) in the sorafenib and control groups, respectively. Concomitant mutations with top 5 occurrence were NPM1 (32%), DNMT3A (16%), TET2 (14%), CEBPA (10%), and IDH2 (7%). The concomitant genetic patterns with ≥1% occurrence are shown in Fig. 2. The top 20 most frequently detected gene mutations were tested for pair-wise mutual exclusivity and co-occurrence in patients with next generation sequencing (NGS) data (Supplementary Figs. S2, S3). A total of 11 pairs were statistically significant for mutual exclusivity after adjustment for false discovery rate (FDR) (Supplementary Fig. S2). For co-occurrence, 11 pairs were significant for independent test, but none remained significant after FDR adjustment (Supplementary Fig. S2).

### Survival, relapse, and GVHD

**Fig. 3:**
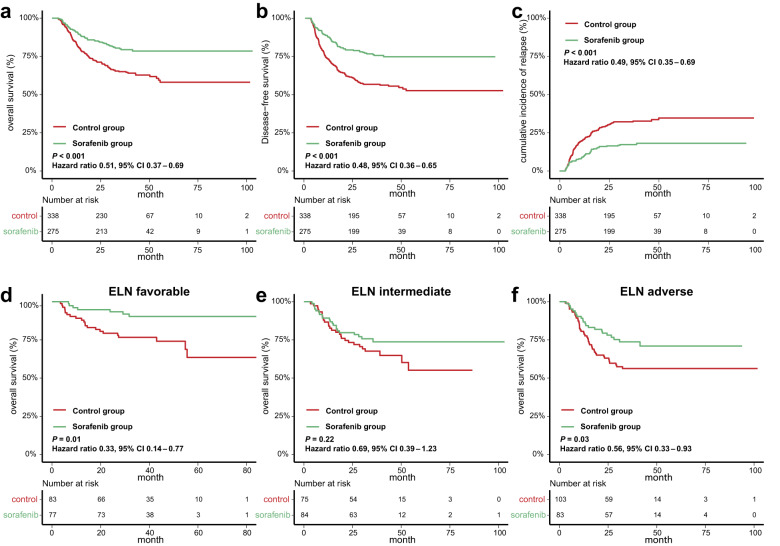
Comparisons of outcomes between the sorafenib and control groups in the whole cohort and ELN risk subgroups. **a** Overall survival, **b** disease-free survival, and **c** cumulative incidence of relapse in the sorafenib and control groups in the whole population. Overall survival of patients with (**d**) favorable, (**e**) intermediate, and (**f**) adverse ELN risk in the sorafenib and control groups

The median follow-up was 36.5 (IQR, 25.2–44.7) months post-transplantation. The 3-year overall survival (OS) was 79.6% (95% CI 74.8%–84.6%) and 65.2% (95% CI 60.3%–70.6%), (HR 0.50, 95% CI 0.37–0.69; *P* < 0.001) (Fig. 3a), and disease-free survival (DFS) was 75.8% (95% CI 70.8%–81.1%) and 57.5% (95% CI 52.4%–63.1%) (HR 0.48, 95% CI 0.36–0.64; *P* < 0.001) in the sorafenib and control groups, respectively (Fig. 3b). The 3-year cumulative incidence of relapse (CIR) was 17.2% (95% CI 13.0%–22.0%) in the sorafenib group and 31.8% (95% CI 26.9%–36.8%) (HR 0.49, 95% CI 0.35–0.69; *P* < 0.001) in the control group (Fig. 3c). We further divided patients into four subgroups based on use of sorafenib pre- and post-transplantation: non-sorafenib pre-transplantation and post-transplantation (group A); sorafenib pre-transplantation only (group B); sorafenib post-transplantation only (group C); and sorafenib pre- and post-transplantation both (group D) for subgroup analyses. Significant differences were observed in OS, DFS, and relapse among groups A-D (Supplementary Fig. S4a–c). Group B, C and D all had superior OS, DFS and CIR compared to group A (Supplementary Fig. S5a). Group C and D both had superior DFS compared to group B, and Group D had superior CIR compared to group B. No significant difference was observed between group C and D (Supplementary Fig. S5a). Multivariate COX analysis revealed that use of sorafenib pre- and post-transplantation both were associated with improved OS (HR 0.73, 95% CI 0.54–0.99; *P* = 0.04 and HR 0.52, 95% CI 0.38–0.72; *P* < 0.001, respectively), DFS (HR 0.71, 95% CI 0.54–0.94; *P* = 0.02 and HR 0.50, 95% CI 0.37–0.67; *P* < 0.001, respectively), and relapse (HR 0.69, 95% CI 0.50–0.94; *P* = 0.02 and HR 0.50, 95% CI 0.35–0.70; *P* < 0.001, respectively) (Table 2).

**Table 2:** Univariable and multivariate analyses for overall survival, disease–free survival, and relapse

	Overall survival^a^		Disease–free survival^a^		Cumulative incidence of relapse^b^	
	Univariate HR (95% CI); p	Multivariate HR (95% CI); p	Univariate HR (95% CI); p	Multivariate HR (95% CI); p	Univariate HR (95% CI); p	Multivariate HR (95% CI); p
Sex, male vs female	1.15 (0.86–1.54); 0.35	–	1.10 (0.84–1.43); 0.50	–	1.08 (0.79–1.47); 0.65	–
Age, >36 years vs ≤36 years	1.24 (0.92–1.66); 0.16	–	1.19 (0.91–1.56); 0.19	–	1.11 (0.81–1.52); 0.51	–
WBC, >53 × 10^9^/L vs ≤ 53 × 10^9^/L	1.09 (0.81–1.46); 0.59	–	1.10 (0.84–1.44); 0.48	–	1.09 (0.80–1.49); 0.60	–
Transplant modality	0.70	–	0.56	–	0.37	–
MSD	1	–	1	–	1	–
MUD	1.34 (0.67–2.67); 0.41	–	1.39 (0.76–2.55); 0.28	–	1.61 (0.83–3.13); 0.16	–
HID	1.09 (0.73–1.62); 0.67	–	1.08 (0.76–1.53); 0.67	–	1.15 (0.77–1.72); 0.51	–
ELN risk stratification	0.0052	0.044	0.00053	0.0073	0.00072	0.0018
Favorable	1	1	1	1	1	1
Intermediate	1.77 (1.12–2.81); 0.015	1.78 (1.11–2.84); 0.016	1.85 (1.21–2.82); 0.0047	1.90 (1.23–2.92); 0.0036	1.96 (1.18–3.27); 0.010	2.04 (1.23–3.41); 0.0061
Adverse	2.20 (1.42–3.41); 0.00040	1.87 (1.16–3.03); 0.011	2.19 (1.46–3.28); 0.00014	2.11 (1.35–3.30); 0.0011	2.38 (1.46–3.86); 0.00048	2.40 (1.47–3.90);0.00043
Unknown	1.89 (1.16–3.09); 0.011	1.26 (0.60–2.66); 0.55	2.34 (1.51–3.63); 0.00015	2.05 (1.00–4.21); 0.052	2.81 (1.68–4.70); <0.0001	2.56 (1.53–4.29); 0.00037
Allelic ratio, ≥ 0.5 vs < 0.5	1.66 (1.18–2.34); 0.0040	1.35 (0.91–1.99); 0.13	1.41 (1.30–1.94); 0.032	1.09 (0.76–1.55); 0.64	1.29 (0.89–1.86); 0.18	–
Pre-transplantation sorafenib, used vs not used	0.74 (0.55–0.99); 0.043	0.73 (0.54–0.99); 0.041	0.70 (0.54–0.92); 0.011	0.71 (0.54–0.94); 0.015	0.68 (0.49–0.93); 0.015	0.69 (0.50–0.94); 0.019
Post-transplantation sorafenib, used vs not used	0.50 (0.37–0.69); <0.0001	0.52 (0.38–0.72); <0.0001	0.48 (0.36–0.64); <0.0001	0.50 (0.37–0.67); <0.0001	0.47 (0.33–0.66); <0.0001	0.50 (0.35–0.70); 0.00062

^a^Results of COX regression analyses

^b^Results of competing risk regression analyses

A total of 267 patients experienced acute GVHD (aGVHD), including 126 in the sorafenib group and 141 in the control group (*P* = 0.31). The 3-year cumulative incidence of chronic GVHD (cGVHD) was 49.8% (95% CI 43.7%–55.7%) in the sorafenib group and 46.4% (95% CI 40.9%–51.6%) (HR 1.08, 95% CI 0.86–1.36; *P* = 0.52) in the control group (Supplementary Fig. S6).

### Effects of sorafenib on survival based on Genetic patterns

Based on ELN 2017 risk stratification, the results showed that sorafenib post-transplantation significantly improved OS in the favorable (HR 0.33, 95% CI 0.14–0.77; *P* = 0.01) and adverse (HR 0.56, 95% CI 0.33–0.93; *P* = 0.03) risk groups but not in the intermediate risk group (HR 0.69, 95% CI 0.39–1.23; *P* = 0.22) (Fig. 3d–f). Subgroup analyses showed significant differences in OS among groups A-D in patients with favorable and adverse risk, but not in patients with intermediate risk (Supplementary Fig. S4d–f). Compared to group A, group C had superior OS in patients with favorable (HR 0.16, 95% CI 0.04–0.71; *P* = 0.02) and adverse (HR 0.47, 95% CI 0.22–0.99; *P* = 0.05) risk, and group D had superior OS in patients with adverse (HR 0.43, 95% CI 0.22–0.83; *P* = 0.01) risk (Supplementary Fig. S5b). No significant difference was observed among groups B, C and D in patients with favorable, intermediate, or adverse risk (Supplementary Fig. S5b). ELN risk subgroups were further dissected by different genetic patterns. The OS of the sorafenib and control groups was compared in the 5 largest genetic pattern subgroups of each ELN risk level (Supplementary Fig. S7). Subgroup details were shown in Table S2. Except the Adverse-Cytogenetics subgroup (HR 0.31, 95% CI 0.12–0.78; *P* = 0.01) who benefitted significantly from sorafenib maintenance post-transplantation, no significant difference was observed in other subgroups (Supplementary Fig. S7). For cytogenetic risk, the results showed that sorafenib post-transplantation maintenance in patients with intermediate (HR 0.57, 95% CI 0.40–0.81; *P* = 0.001) and adverse (HR 0.33, 95% CI 0.13–0.82; *P* = 0.02) cytogenetics had a significant benefit on OS, while patients with favorable (HR 0.15, 95% CI 0.02–1.16; *P* = 0.07) cytogenetics had the same trend (Supplementary Fig. S8).

**Fig. 4:**
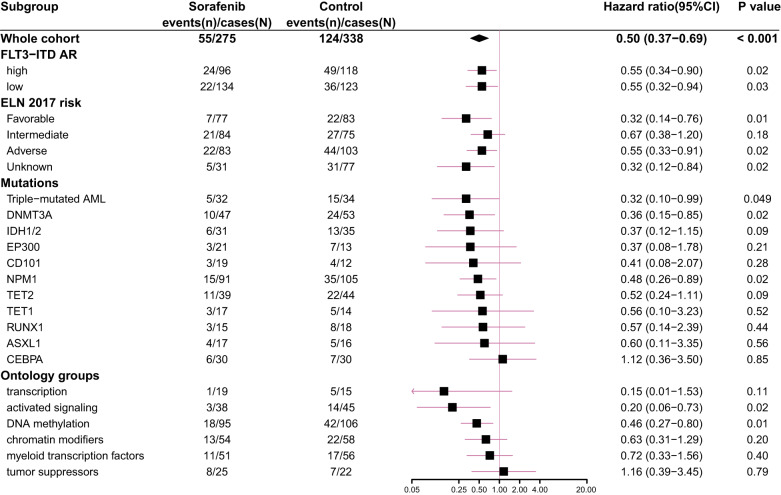
Multivariate COX results of sorafenib maintenance post-transplantation effects on the subgroups with different ELN risk groups, FLT3-ITD AR, and genetic patterns

Based on concomitant mutations, we analyzed gene mutations in patients with >5% occurrence and triple-mutated (co-occurring NPM1, DNMT3A and FLT3-ITD mutations) (Fig. 4). Multivariate COX regression results showed that patients with mutated NPM1 (HR 0.48, 95% CI 0.26–0.89; *P* = 0.02), DNMT3A (HR 0.36, 95% CI 0.15–0.85; *P* = 0.02), and triple-mutated (HR 0.32, 95% CI 0.10–0.99; *P* = 0.049) in the sorafenib group had superior OS than the control group, and those with TET2 (HR 0.52; 95% CI 0.24–1.11; *P* = 0.09) and IDH1/2 (HR 0.37; 95% CI 0.12–1.15; *P* = 0.09) in the sorafenib group showed a trend of improved OS. There was no significant difference in OS of patients with CEBPA (HR 1.12; 95% CI 0.36–3.50; *P* = 0.85), ASXL1 (HR 0.60; 95% CI 0.11–3.35; *P* = 0.56), TET1 (HR 0.56; 95% CI 0.10–3.23; *P* = 0.52), CD101 (HR 0.41; 95% CI 0.08–2.07; *P* = 0.28), EP300 (HR 0.37; 95% CI 0.08–1.78; *P* = 0.21), and RUNX1 (HR 0.57; 95% CI 0.14–2.39; *P* = 0.44) mutations between the two groups (Fig. 4).

We next explored mutations in 6 gene ontology groups (“activated signaling”, “DNA methylation”, “transcription”, “chromatin modifiers”, “tumor suppressor”, and “myeloid transcription factors”) (Supplementary Table 3). The results are shown in Fig. 4. Patients carrying mutated “activated signaling” (HR 0.20, 95% CI 0.06–0.73; *P* = 0.02) and “DNA methylation” (HR 0.46, 95% CI 0.27–0.80; *P* = 0.01) genes with sorafenib maintenance post-transplantation had superior OS than those with control, and those with “transcription” genes showed the superior trend (HR 0.15, 95% CI 0.01–1.53; *P* = 0.11), but not those with “tumor suppressors” (HR 1.16, 95% CI 0.39–3.45; *P* = 0.79), “myeloid transcription factors” (HR 0.72, 95% CI 0.33–1.57; *P* = 0.40), and “chromatin modifiers” genes (HR 0.63, 95% CI 0.31–1.29; *P* = 0.20) (Fig. 4).

### Effects of sorafenib on FLT3-ITD AR

Based on FLT3-ITD AR, 471 patients obtained FLT3-ITD AR data, with 214 FLT3-ITD^high^ (AR ≥ 0.50) and 257 FLT3-ITD^low^ (AR < 0.50).^4^ Sorafenib maintenance post-transplantation improved OS in patients with FLT3-ITD^high^ (HR 0.55, 95% CI 0.34–0.90; *P* = 0.02) and FLT3-ITD^low^ (HR 0.54, 95% CI 0.32–0.92; *P* = 0.02) (Supplementary Fig. S9a, b). In subgroup analyses, significant difference was found in FLT3-ITD^high^ (*P* = 0.004) but not FLT3-ITD^low^ (*P* = 0.07) patients among groups A-D (Supplementary Fig. S9c, d). In FLT3-ITD^high^ patients, compared to group A, group B (HR 0.48, 95% CI 0.26–0.87; *P* = 0.02), C (HR 0.49, 95% CI 0.26–0.94; *P* = 0.03) and D (HR 0.33, 95% CI 0.16–0.65; *P* = 0.001) all had improved OS, while in FLT3-ITD^low^, significant difference was found in group C compared with group A (HR 0.39, 95% CI 0.18–0.85; *P* = 0.02), a trend of improved OS was also found in group D (HR 0.54, 95% CI 0.28–1.04; *P* = 0.07) (Supplementary Fig. S10). No significant difference was observed among groups B, C and D in either FLT3-ITD^high^ or FLT3-ITD^low^ patients (Supplementary Fig. S10).

### The association between tumor suppressor and myeloid transcription factor mutations and sorafenib sensitivity, and the mutational evolution of FLT3-ITD AML

Since we observed association between concomitant “tumor suppressor”, and “myeloid transcription factors” mutations and decreased sorafenib response, we next explored whether concomitant mutations associated with sorafenib sensitivity using bulk RNA-seq data of bone marrow cells from FLT3-ITD AML patients. A total of 197 FLT3-ITD AML patients with available RNA-seq and mutational data was collected from the BeatAML and TCGA-LAML datasets.

Patients were grouped according to concomitant mutations (Supplementary Fig. S11a), and the half maximal inhibitory concentration (IC50) of sorafenib was compared among groups (Supplementary Fig. S11b). Interestingly, group 1 had the highest IC50 among the 4 groups, while group 2 had the lowest, and group 0 and group 3 had an intermediate IC50 as compared to the other 2 groups (Supplementary Fig. S11b). Both group 2 (*P* = 0.007) and group 0 (*P* = 0.02) had significantly lower IC 50 than group 1(Supplementary Fig. S11b).

Since group 1 showed higher sorafenib resistance than group 2, we next performed differential analyses between the two groups to discover the possible mechanisms for this difference (Supplementary Fig. S11c). A total of 2245 significantly differentially expressed genes was observed, with 914 highly expressed in group 1 and 1331 in group 2 (Supplementary Fig. S11c). Notably, the highly expressed genes in group 1 enriched in JAK-STAT pathway, MAPK cascade, and ERK cascade (Supplementary Fig. S11d). Similar results were also obtained in gene set variation analysis (GSVA) (Supplementary Fig. S11e). Given that the alternative activation of FLT3 downstream signaling like STATs, mTOR, and MAPK was an important mechanism of sorafenib resistance, we hypothesized that concomitant tumor suppressors or myeloid transcription factors mutations in FLT3-ITD AML drove sorafenib resistance via activating FLT3 downstream signaling.

TP53 was chosen as a representative to test this hypothesis. We electrotransfected pLenti-CMV-TP53(R248Q)-GFP-Puro (TP53 mut), pLenti-CMV-TP53-GFP-Puro (TP53 WT), and pLenti-CMV-GFP-Puro (EV) into MV411 cells (Supplementary Fig. S11f). As shown by the results of CCK-8 assay, the TP53 mut groups had significantly higher cell viability after treated with sorafenib for 24 and 48 h compared to the TP53 WT and EV groups (Supplementary Fig. S11g). Addition of STAT3 inhibitor significantly reduced sorafenib resistance in TP53 mut groups of at both 24 and 48 h (Supplementary Fig. S11h). Taking together, these data suggested that TP53 mutation might drive sorafenib resistance via activating the JAK-STAT signaling pathway in FLT3-ITD AML.

Finally, we explored the mutational evolution of FLT3-ITD AML. The genetic patterns of patients who relapsed with NGS data at both diagnosis and relapse were analyzed (Supplementary Fig. S12). A total of 21 patients were analyzed, including 6 in the sorafenib group and 15 in the control group. Eight patients lost FLT3-ITD at relapse, including 3 in the sorafenib group and 5 in the control group. Five patients acquired “activated signaling” mutations at relapse (2 acquired NRAS, 2 acquired KRAS, and 1 acquired KIT), among which 4 received sorafenib (3 received pre-transplantation sorafenib, and 1 received both pre- and post-transplantation sorafenib) (Supplementary Fig. S12). Of note, 3 patients in the sorafenib and 1 patient in the control group acquired FLT3 tyrosine kinase domain (TKD) mutation at relapse. However, none of these patients received NGS and were thus not analyzed.

## Discussion

In this study, we characterized the mutational landscape and its clinical significance in 613 patients with FLT3-ITD AML evaluating the effect of sorafenib maintenance post-transplantation, suggesting a broad beneficial effect of sorafenib across various genetic patterns. Meanwhile, we further confirmed the results of previous retrospective and prospective studies,^9,14,16,20,31,32^ in which sorafenib pre- or post-transplantation both could improve survival in patients with FLT3-ITD AML without increasing the risk of GVHD, especially sorafenib maintenance post-transplantation.

The effect of FLT3 inhibitors is correlated with genetic patterns in patients receiving chemotherapy.^6,18,28,29^ However, the role of FLT3 inhibitors in different genetic patterns remains unclear. An analysis of RCT data revealed that FLT3-ITD AML patients with all ELN cytogenetic risk groups might benefit from midostaurin, while another report about midostaurin reported an improved relapse rate in the intermediate risk group only.^6,33^ In this study, our results showed that sorafenib post-transplantation could improve survival of FLT3-ITD AML patients with both favorable and adverse risk, but did not in those with intermediate risk. The results require to be verified by large-sample clinical studies.

As for gene mutations, FLT3-mutated AML patients with NPM1 receiving chemotherapy were reported to benefit from sorafenib, gilteritinib and midostaurin.^18,28,29^ Recently, Smith et al. reported that gilteritinib improved OS in the patients with co-occurring mutations on NPM1, DNMT3A and triple-mutated, but could not overcome those with TP53.^28^ These results came mainly from the patients undergoing chemotherapy. In this study, we focused on the effect of sorafenib on the outcomes of FLT3-ITD AML patients undergoing allo-HSCT. The results of our large-scale cohort revealed that most concomitant genetic patterns showed a trend of favoring the sorafenib group. Patients with co-occurring NPM1, DNMT3A and triple-mutated benefited significantly from sorafenib maintenance post-transplantation, which were in consistent with the results reported in the chemotherapy setting.^18,28^ However, those with co-occurring CEBPA mutation did not benefit from sorafenib maintenance post-transplantation. One reasonable interpretation of this finding was that the beneficial effects of sorafenib might be overwhelmed by allo-HSCT in patients with CEBPA mutation.^34^ Although these findings were very interesting and provided basis for further studies, they had to be interpreted cautiously due to small numbers.

The effects of FLT3 inhibitors on FLT3-ITD AML patients carrying abnormalities on different concomitant gene ontology groups were rarely reported. Recently, Jahn et al. reported that midostaurin might improve OS and DFS of FLT3-mutated AML patients with chromatin-spliceosome mutations.^29^ Smith et al. reported that gilteritinib might improve OS in FLT3-mutated patients carrying mutated DNA methylation/hydroxymethylation mutations, while it did not improve the outcome of those with mutated RTK/Ras signaling genes.^28^ In this study, we found that the patients with “activated signaling” and “DNA methylation” mutations might benefit from sorafenib. Despite the different population and intervention of both studies, the pharmacological characteristics between sorafenib and gilteritinib might help explain this difference.^2,30,35^ As a first-generation FLT3 inhibitor, besides directly blocking the activity of FLT3, sorafenib had also potential inhibition effect on other tyrosine kinase receptors like PDGFR and KIT, and downstream molecules of FLT3 signaling such as KRAS and BRAF.^2,30^ On the other hand, we also found that patients with “tumor suppressors” and “myeloid transcription factors” mutations did not response significantly to sorafenib. For tumor suppressors, wild type p53 protein was reported to inhibit the phosphorylation of STAT3, and the transcriptional activity of both STAT3 and STAT5, which could be lost through TP53 mutation.^36,37^ Similarly, our data showed that TP53 mutation might increase sorafenib resistance via activating STAT3. PHF6 mutation might induce sorafenib resistance via upregulating JAK-STAT signaling and increasing the ubiquitination of the p53 protein.^38,39^ As for myeloid transcription factors, CEBPA mutations were also associated with activated JAK-STAT signaling, especially those with biallelic mutation.^40^ (Supplementary Fig. S13). Nevertheless, although several different gene mutations were shown to correlate with upregulated JAK-STAT signaling, the exact role JAK-STAT pathway played in the disease development and sorafenib resistance should be discussed in a mutation-by-mutation manner. Further basic studies are warranted before clinical attempts of combing sorafenib and other targeted therapies (STAT inhibitors, etc) in certain genetic subgroups of FLT3-ITD AML could be made.

The role of FLT3 inhibitors in FLT3-ITD^high^ AML has been widely established, while controversial results are reported regarding the effects of FLT3 inhibitors on FLT3-ITD^low^ AML patients receiving chemotherapy.^18,41^ In our study, sorafenib pre-transplantation improved OS in FLT3-ITD^high^ AML patients, but it did not in FLT3-ITD^low^ AML. This concorded with that reported by Abou Dalle et al. in the patients receiving chemotherapy.^18^ In contrast, we found that sorafenib maintenance post-transplantation might improve the survival of both FLT3-ITD^high^ and FLT3-ITD^low^ AML patients, suggesting that sorafenib maintenance post-transplantation was required in FLT3-ITD^low^ AML.

There are currently few data on the mutational evolution of FLT3-ITD AML in the context of FLT3 inhibitors. Smith et al. reported that 9 of 39 FLT3-mutated patients receiving gilteritinib lost FLT3 mutation at relapse, and 5 patients acquired the FLT3 F691L “gatekeeper” mutattion.^28^ Schmalbrock et al. reported a 46% rate of losing FLT3 mutation at relapse in patients receiving midostaurin, compared to 19% in those who did not.^42^ In our study, 8 in 21 patients lost FLT3-ITD at relapse. Four additional patients (3 in the sorafenib and 1 in the control group) acquired FLT3-TKD. The sorafenib group seemed to have slightly higher rate of losing FLT3-ITD (3 in 6) compared to the control group (5 in 15). For activated signaling genes, in the study of Smith et al., 16 in 39 patients receiving gilteritinib acquired NRAS, KRAS or PTPN11.^28^ However, in the study of Schmalbrock et al., only 4 in 54 patients receiving midostaurin acquired activated signaling genes at relapse.^42^ Our data showed that 5 in 21 patients acquired activated signaling genes at relapse, most of whom received sorafenib, either pre- or post-transplantation. The different mutational evolution patterns might be related to the different pharmacological characteristics of FLT3 inhibitors. A study by Alotaibi et al. showed that FLT3-mutated patients might have different patterns of mutational evolution between those who received type I and type II FLT3 inhibitors.^43^ However, considering the diverse study settings and relatively small sample sizes of current studies, further studies were warranted to confirm these findings.

Our study had a few limitations. Patients included in this study was a pooled population from four prospective and retrospective cohort with differences in details such as sorafenib administration. Also, despite the large-scale of this study, the number of patients in certain genetic pattern subgroups was still too small for a statistically significant result.

In conclusion, our study identifies the response of genetic patterns to sorafenib, and further confirms the role of sorafenib in FLT3-ITD AML patients undergoing allo-HSCT. This study provides new viewpoints for the optimal use of sorafenib in FLT3-ITD AML patients undergoing allo-HSCT based on genetic patterns.

## Materials and methods

### Study design and participants

In this multicenter, cohort study, the study population came from our four studies, including a prospective observational study (NCT03620955), an RCT (NCT02474290),^9^ and two retrospective studies.^14,44^ Patients met the following criteria were enrolled: (a) aged 18–65 years; (b) diagnosed with FLT3-ITD AML; (c) underwent first allo-HSCT. Patients were excluded from the study if they met any of the following criteria: (a) diagnosed with acute promyelocytic leukemia; (b) received other FLT3 inhibitors pre-transplantation or as maintenance therapy post-transplantation; (c) failed to achieve CRc, relapsed or died within 90 days post-transplantation; (d) initiated sorafenib maintenance after 180 days post-transplantation; (e) lack of concomitant genomic data. The diagnosis and risk stratification of AML were according to the guideline of the National Comprehensive Cancer Network and the 2017 ELN AML recommendation.^4^ CRc comprised complete CR, CR with incomplete platelet recovery, and CR with incomplete hematological recovery.^9^ CRc and partial remission (PR) were defined as per the 2017 ELN AML recommendations.^4^ NR was defined as achieving the standards of neither CRc nor PR. The protocol was reviewed and approved by the institutional review board of each participating center and was conducted in accordance with the Declaration of Helsinki. Written informed consent was obtained from the donors and recipients before the initiation of the study.

### Procedures

Patients who received sorafenib maintenance post-transplantation for >4 weeks were allocated to the sorafenib group, and those who did not receive sorafenib maintenance post-transplantation or received sorafenib maintenance for <4 weeks were allocated to the control group. Some patients received pre-transplantation sorafenib as part of the induction therapy combined with chemotherapy, or post-remission maintenance therapy, or both. Patients who used sorafenib for at least 4 weeks before allo-HSCT were considered receiving sorafenib pre-transplantation, regardless of the context. Those who did not receive sorafenib, or received for less than 4 weeks pre-transplantation were considered not receiving sorafenib pre-transplantation. For patients who received sorafenib, the initial dose was 400 mg twice daily, and adjusted according to tolerance of patients under the guidance of physicians or researchers. For patients from the prospective studies, the methods for dose modification were reported previously.^9^

Cytogenetic analyses were performed with the Giemsa and reverse banding techniques, and FISH. Gene mutations were tested using direct sequencing and a 167-gene NGS (Table S4). PCR and direct sequencing, or NGS was used to test FLT3-ITD. FLT3-ITD positive was judged using the threshold of mutant-to-wild-type AR ≥ 0.03.

### Outcomes

The endpoints in this study were OS, DFS, relapse, and GVHD in the whole cohort and OS in genetic pattern subgroups. OS was defined as duration since transplantation till death of any cause. DFS was defined as duration since transplantation till the occurrence of relapse, or death from any cause. Relapse was defined as the existence of any of the following: (a) reappearance of leukemic blasts in the peripheral blood; (b) bone marrow blasts ≥5% as shown by bone marrow aspirate or biopsy specimen which could not be explained by any other reason; (c) reappearance or new appearance of extramedullary leukemia. GVHD, including aGVHD and cGVHD, was defined according to guidelines.^45,46^

### Bioinformatic analyses

Patients with bulk RNA-seq data within the BeatAML and TCGA-LAML datasets was screened for FLT3 mutation status. Counts, clinical, mutational, and drug sensitivity (for the BeatAML dataset) data of FLT3-ITD positive AML patients was downloaded. Mutations were screened according to the mutation data in the cBioPortal database for the TCGA-LAML dataset, and the results from clinical genotyping data or targeted DNA sequencing/whole exome sequencing for the BeatAML dataset.

The DEseq2 method was applied for removing batch effect and differential analyses. The threshold for significant difference of gene transcription level between groups was adjusted *P*-value < 0.05 and log2(fold change) > 1. Differentially expressed genes were used for enrichment analyses. Raw counts matrices were used for GSVA analyses.

The IC50 of sorafenib was extracted from the drug sensitivity data in the BeatAML dataset and compared among groups within cases with available drug sensitivity data.

### Electrotransfection

The cells were passaged 24 h before the electrotransfection to ensure they were in logarithmic growth. Before electrotransfection, the concentration of cells was adjusted to 107/ml using 1640 medium. The pLenti-CMV-TP53(R248Q)-GFP-Puro, pLenti-CMV-TP53-GFP-Puro, or pLenti-CMV-GFP-Puro plasmid (PPL, Nanjing, China) was added to the cell suspension, adjusting the final concentration of plasmids to 20 μg/ml. Electrotransfection was performed using ECM830 (BTX, New York, USA), and confirmed by GFP expression under the IX73 inverted microscope (Olympus, Tokyo, Japan).

### CCK-8 assay

Cells were transferred into 96-well plates (Corning, New York, USA) at a density of 5000 cells per well, and exposed to DMSO (YEASEN, Wuhan, China), 5 μg/ml of sorafenib (MCE, New Jersey, USA), or 5 μg/ml of sorafenib combined with 3 μg/ml of STAT3 inhibitor (MCE, New Jersey, USA) for 0, 24 and 48 h. Cell viability was assessed using the CCK-8 solution (FDbio, Hangzhou, China) at different timepoints and measured using the MB-580 Microplate Analyzer (HEALES, Shenzhen, China).

### Statistical analysis

The statistical analysis for the clinical data was done on September 1, 2022. Continuous variables were presented as mean and standard deviation for those normally distributed, or median and IQR for those non-normally distributed. All categorical variables were presented as frequency and proportion. Comparations of continuous variables between groups were conducted using Mann-Whitney U test, and comparations of categorical variables between groups were performed using ${\chi }^{2}$ test or Fisher’s exact test. The mutual exclusivity and co-occurrence of genetic abnormalities were tested using the discrete independence statistic controlling for observations with varying event rates (DISCOVER) method.^47^

Analyses of OS, DFS, relapse and cGVHD were mainly compared between the sorafenib and control groups. OS and DFS were compared using the Kaplan-Meier analysis with the log-rank test. Cumulative incidences of relapse and cGVHD were calculated and compared using the Fine and Gray model.^48^ Non-relapse mortality was a competing risk for relapse. Relapse and death without cGVHD were competing risks for cGHVD.

HR and 95% CI were calculated using the COX proportional hazards models for OS and DFS, and competing risk regression model for relapse. The following variables were included in the COX or competing risk regression models for the whole cohort: (a) sex (male vs female), (b) age (>36 years vs ≤36 years), (c) white blood cell (WBC) counts at diagnosis (>53 × 10^9^/L vs ≤53 × 10^9^/L), (d) transplant modality (HID, MUD vs MSD), (e) ELN 2017 risk stratification (intermediate, adverse, unknown vs favorable), (f) FLT3-ITD AR (≥0.50 vs <0.50), (g) sorafenib pre-transplantation (used vs not used), and (h) sorafenib post-transplantation (used vs not used). Variables were analyzed using a univariable model respectively at first, and only the variables with p < 0.10 were included into the multi-variable models. Univariable COX regression models were used to analyze the effect of sorafenib usage before and after transplantation (pre-transplantation only, post-transplantation only, both pre- and post-transplantation vs non-sorafenib) on ELN risk groups and FLT3-ITD AR.

All levels of significance were set at two-sided 5% level. All statistical analyses were done using SPSS 25.0 IBM (IBM Corp., Armonk, NY) and R 4.1.0 (R Project for Statistical Computing, Vienna, Austria).

### Supplementary information

Supplementary material

## Data Availability

The RNA-seq, drug sensitivity, and metadata of TCGA-LAML and BeatAML datasets are available from their original source data. For the prospective cohorts (NCT03620955 and NCT02474290), de-identified individual participant data underlying the results reported in this paper, and the study protocol will be made available beginning 9 months after publication of the original paper. For the retrospective cohorts, de-identified individual participant data underlying the results reported in this paper will be made available beginning 9 months after publication of this paper. All requests for data should be made by contacting Prof. Qifa Liu (liuqifa628@163.com), and will be assessed by an independent review committee on a case-by-case basis. After 36 months of publication of each dataset, corresponding data will be available on request to Prof. Qifa Liu, as per the access approach of the data repository unit at Southern Medical University, Guangzhou, China.
