# Supplementary material for: Impact of genetic patterns on sorafenib efficacy in patients with FLT3-ITD acute myeloid leukemia undergoing allogeneic hematopoietic stem cell transplantation: a multi-center, cohort study
Source: Signal Transduct Target Ther. 2023 Sep 14;8:348. doi: 10.1038/s41392-023-01614-1 (PMC10499827; doi:10.1038/s41392-023-01614-1)
Supplement: Supplementary file 1 — Supplementary material [file 41392_2023_1614_MOESM1_ESM.docx]

Supplementary Materials for

Impact of genetic patterns on sorafenib efficacy in patients with FLT3-ITD acute myeloid leukemia undergoing allogeneic hematopoietic stem cell transplantation: a multi-center, cohort study

Ruoyang Shao; Yu Zhang; Jinping He; Fen Huang; Zhiping Fan; Kaibo Yang; Yajing Xu; Na Xu; Yi Luo; Lan Deng; Xi Zhang; Jia Chen; Mingzhe Han; Xudong Li; Sijian Yu; Hui Liu; Xinquan Liang; Xiaodan Luo; Pengcheng Shi; Zhixiang Wang; Ling Jiang; Xuan Zhou; Ren Lin; Yan Chen; Sanfang Tu; Jing Sun; Yu Wang; Qifa Liu; Li Xuan

Correspondence to:

Prof. Li Xuan, MD, Department of Hematology, Nanfang Hospital, Southern Medical University, Guangzhou, 510515, China, Tel: 86-20-62787883, Fax: 8620-62787883, Email: 356135708@qq.com

Prof Qifa Liu MD, Department of Hematology, Nanfang Hospital, Southern Medical University, Guangzhou, 510515, China, Tel: 86-20-62787883, Fax: 8620-62787883, Email: liuqifa628@163.com.

Prof. Yu Wang, MD, Department of Hematology, Peking University People’s Hospital, Beijing, 100044, China, Tel: 86-10-88326666, Fax: 8610-88326666, Email: [ywyw3172@sina.com](mailto:ywyw3172@sina.com)

**This PDF file includes:**

Figures. S1 to S13

Tables S1 to S4


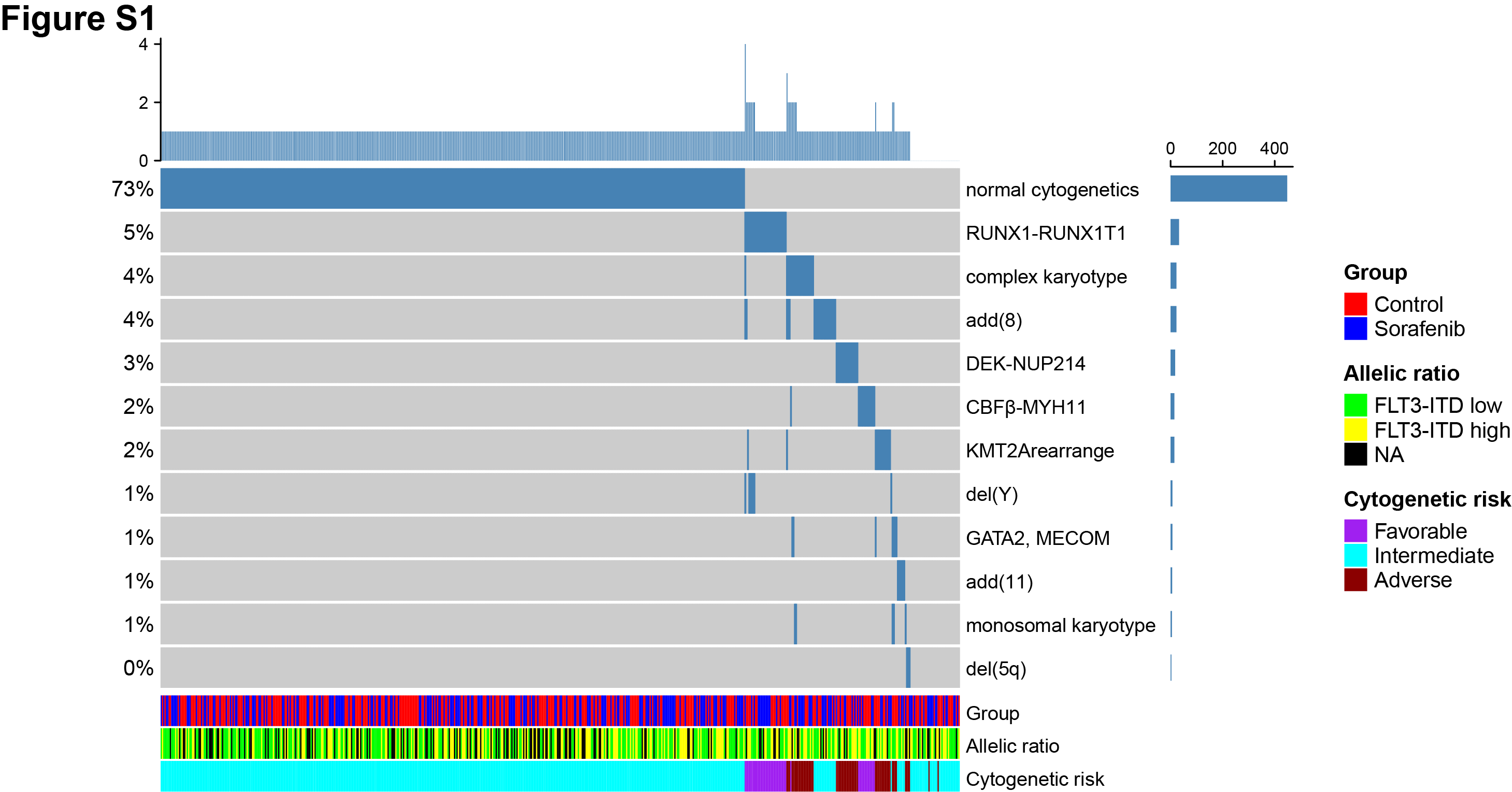


Figure. S1.

**Cytogenetic abnormality patterns of the whole population (Cytogenetic abnormalities detected in** **≥ 0.5% patients).**


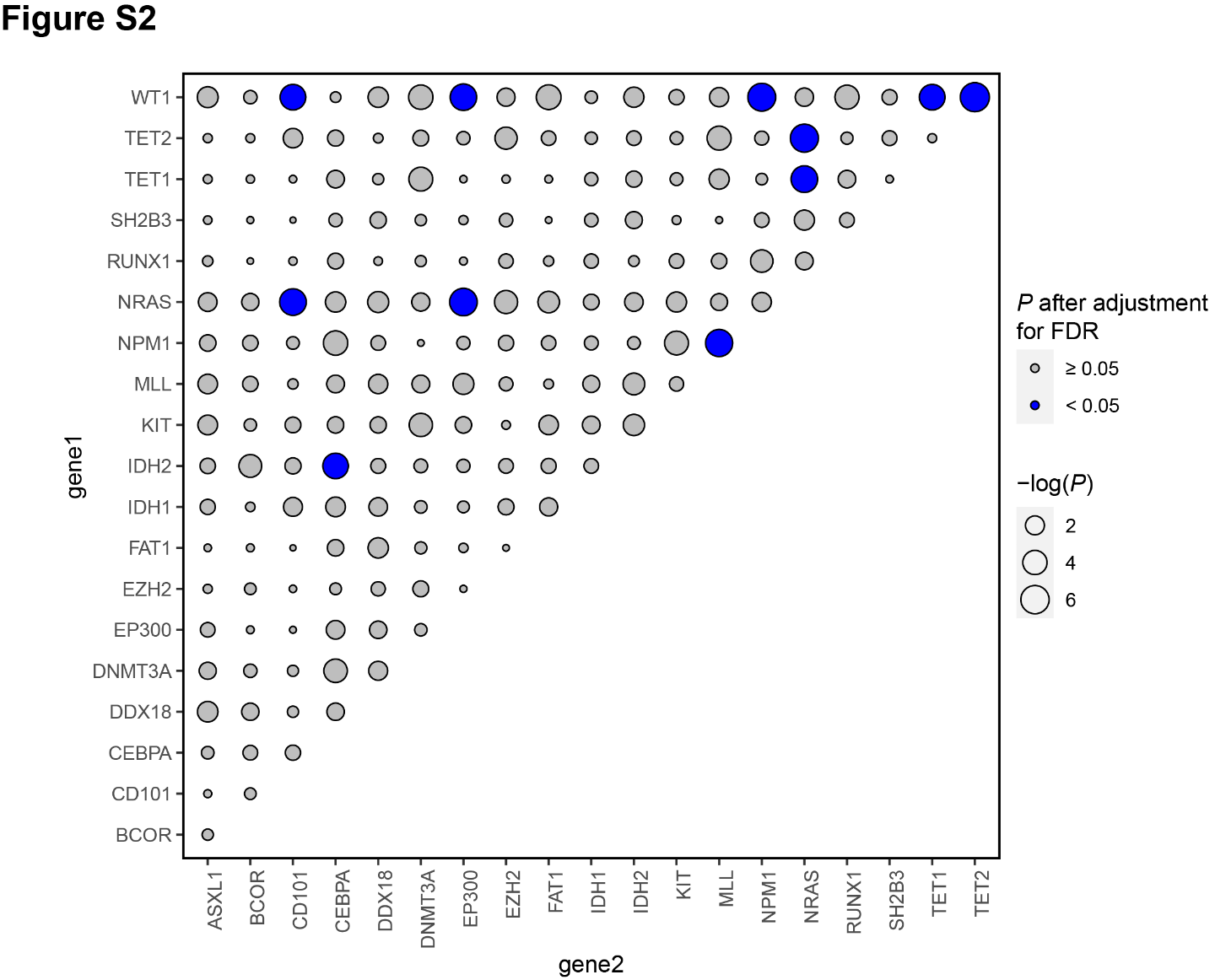


Figure. S2.

**Mutually exclusivity of top 20 most frequently detected mutations.** Each point represents a genomic abnormality pair. Point size indicates p value of independent tests. Color shows p after adjustment for FDR by multiple tests.


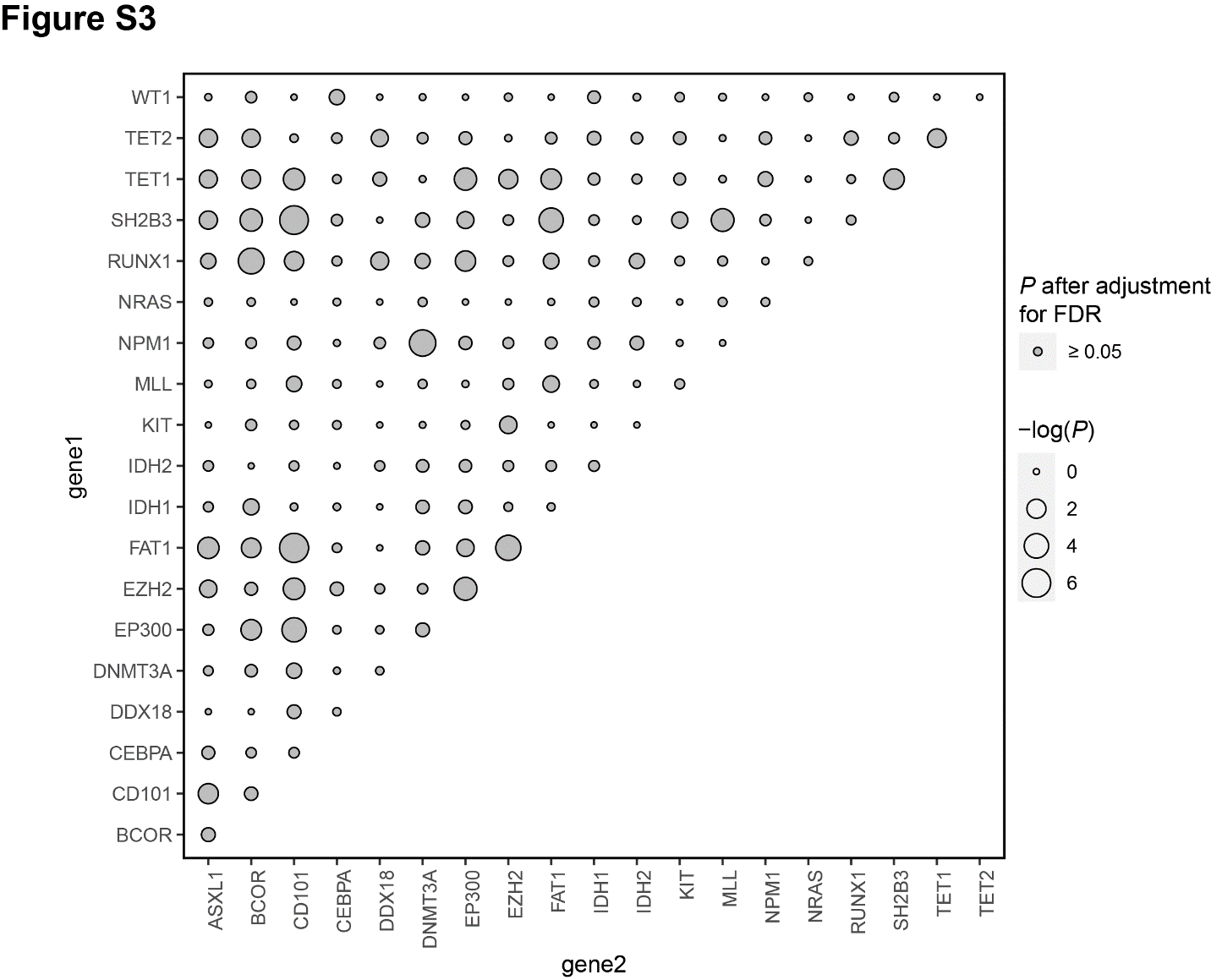


Figure. S3.

**Co-occurrence of top 20 most frequently detected mutations.** Each point represents a genomic abnormality pair. Point size indicates p value of independent test. Color shows p after adjustment for FDR by multiple tests


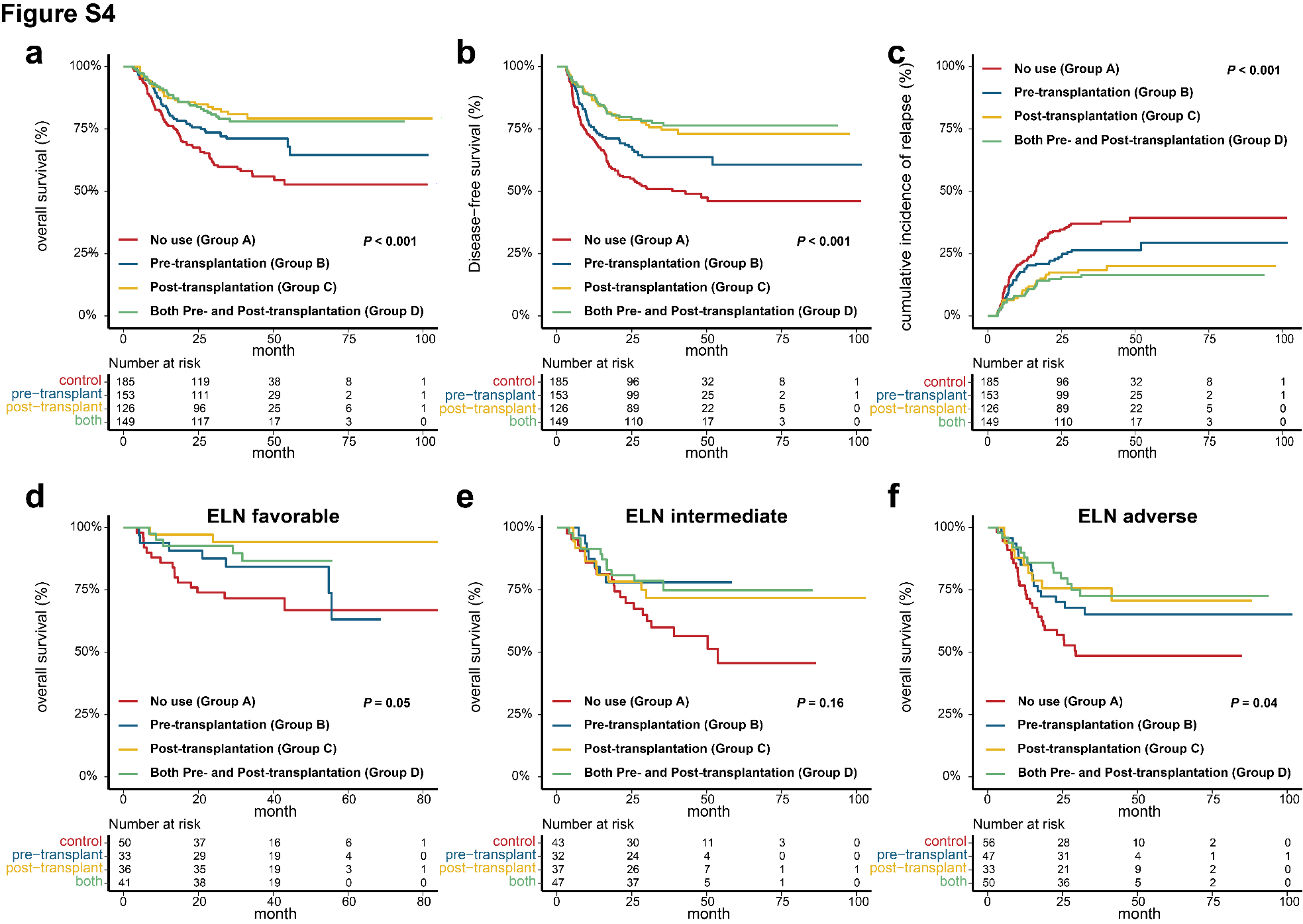


Figure. S4.

**Comparisons of outcomes among groups A-D (based on use of sorafenib pre- and post-transplantation) in the whole cohort and ELN risk subgroups.** (a) Overall survival, (b) disease-free survival and (c) cumulative incidence of relapse among groups A-D in the whole cohort. Overall survival in patients with (d) favorable ELN risk, (e) intermediate ELN risk, and (f) adverse ELN risk among groups A-D.


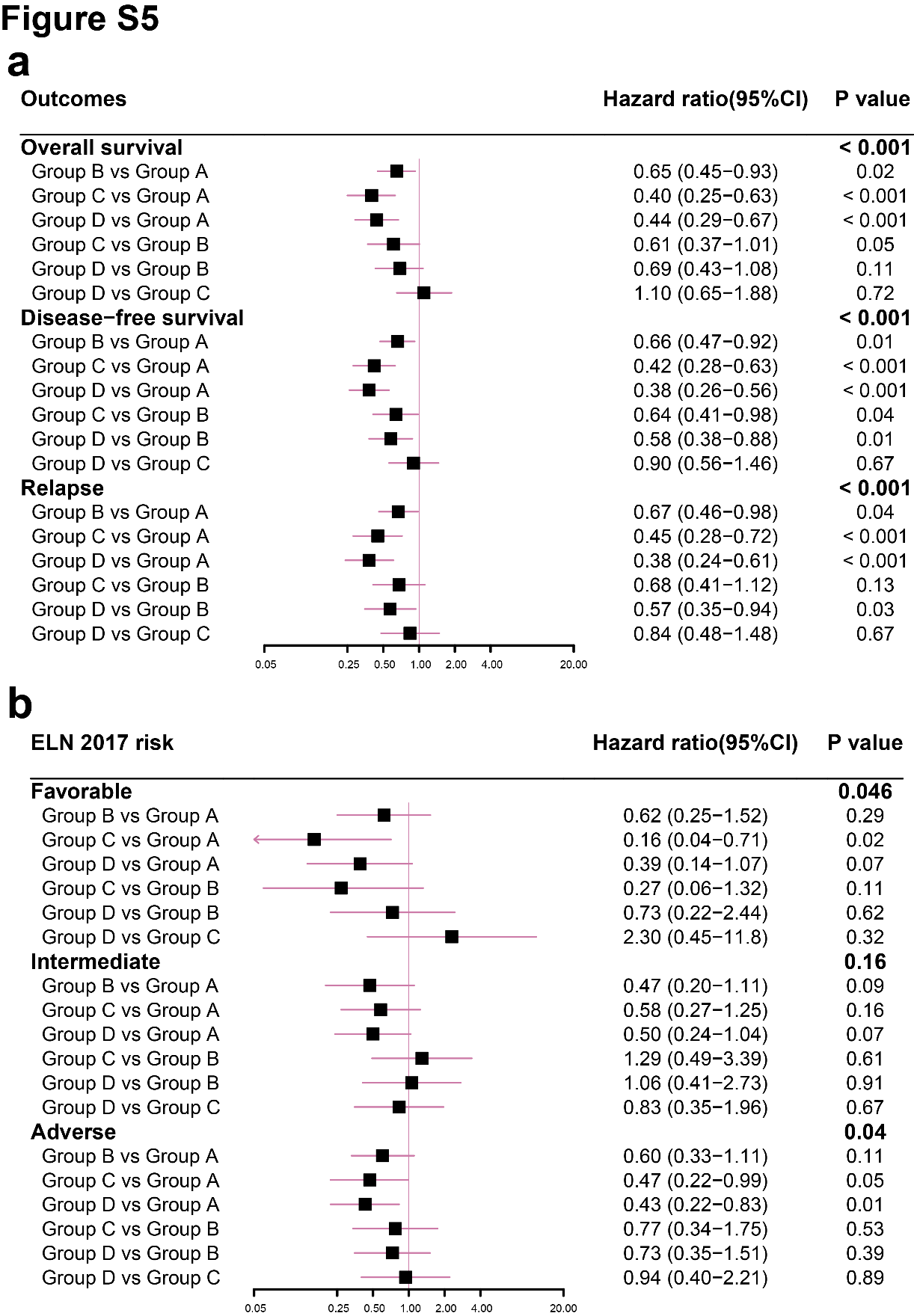


Figure. S5.

**Hazard ratios of (a) OS, DFS and CIR in the whole cohort and (b) OS in ELN risk groups among groups A-D (based on pre- and post-transplantation sorafenib).**


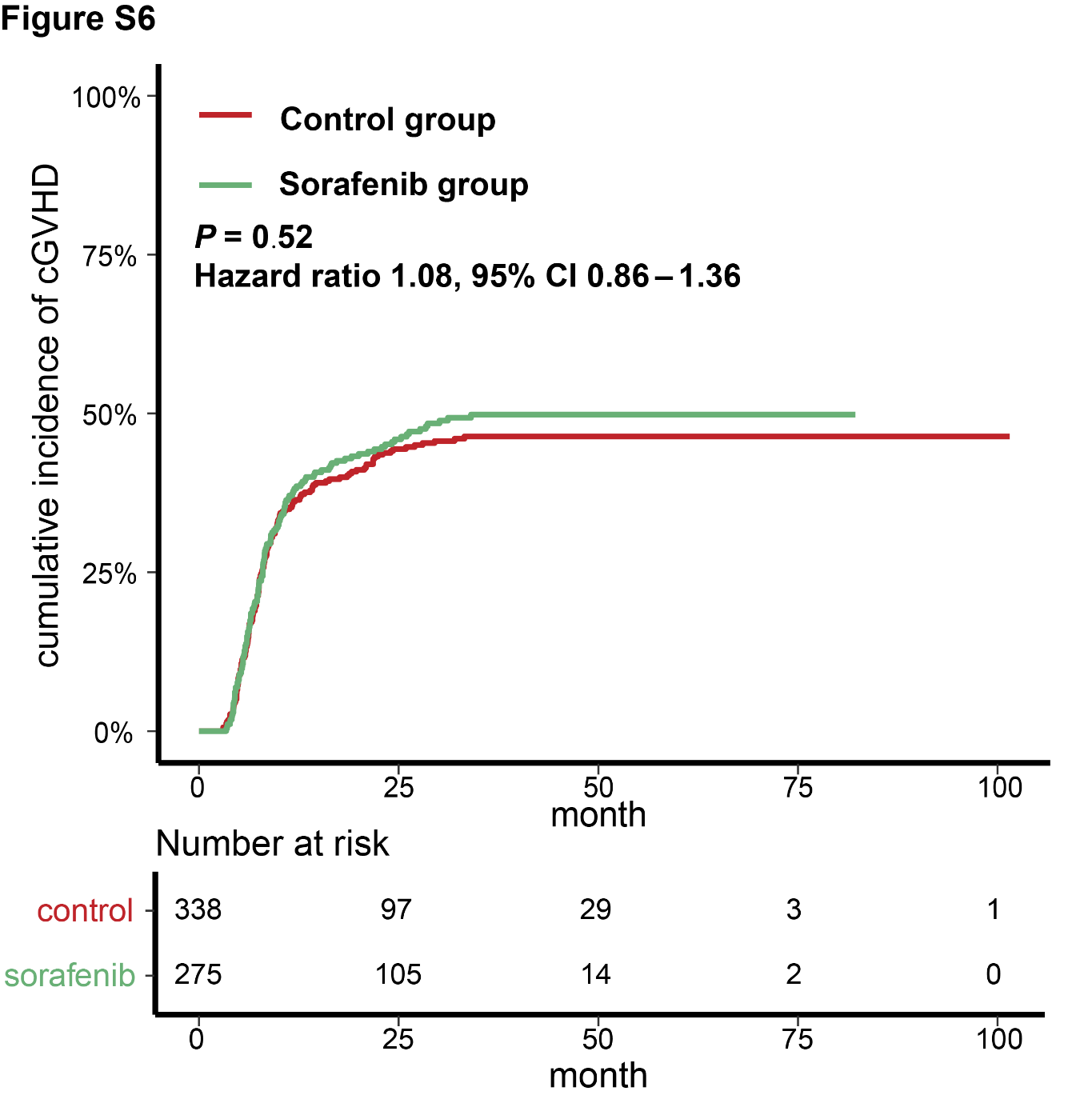


Figure. S6.

**Comparisons of cumulative incidence of cGVHD between the sorafenib and control groups in the whole cohort.**


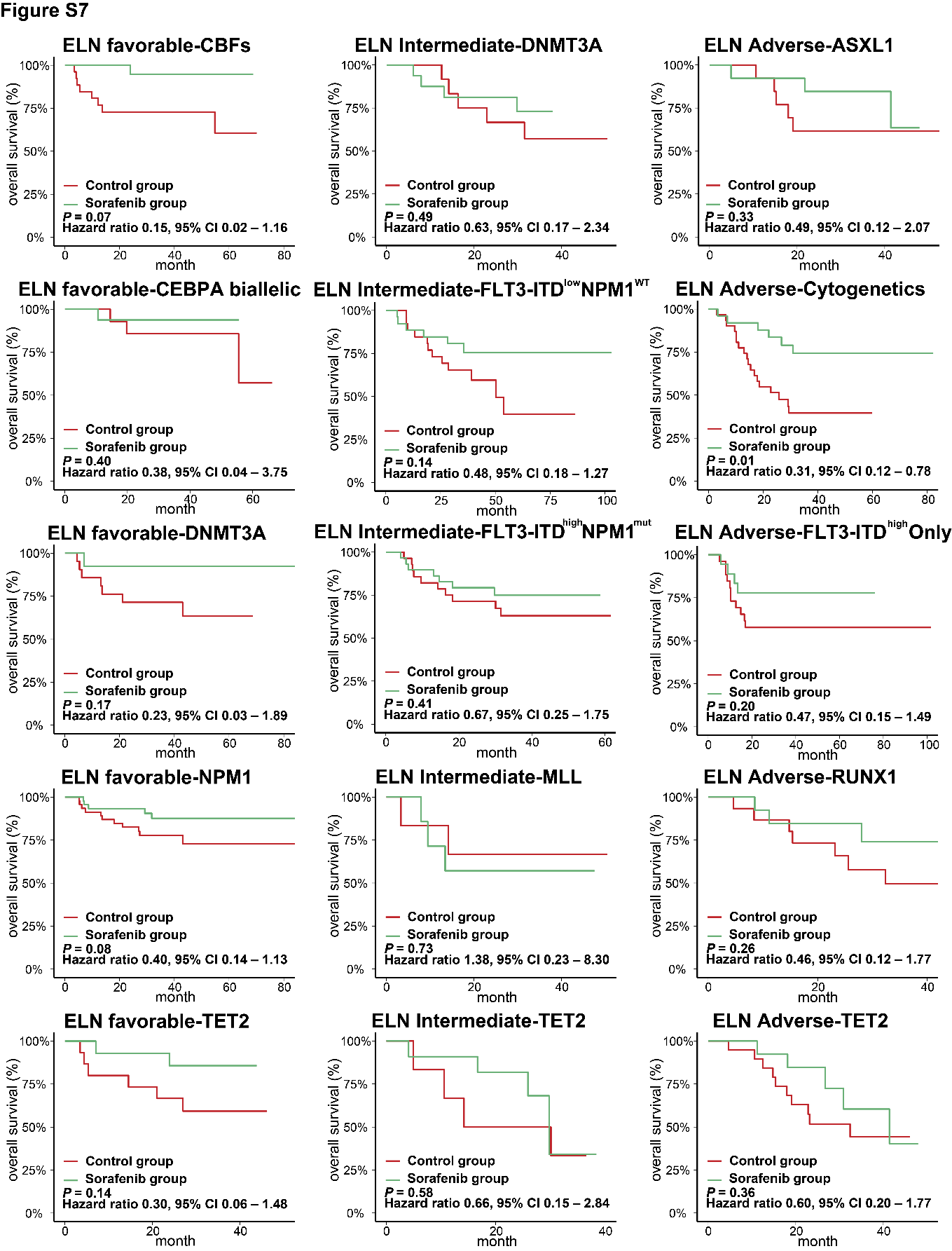


Figure. S7.

**Comparisons of OS between the sorafenib and control groups in the 5 largest genetic subgroups in patients with (a) favorable, (b) intermediate, and (c) adverse ELN risk.**


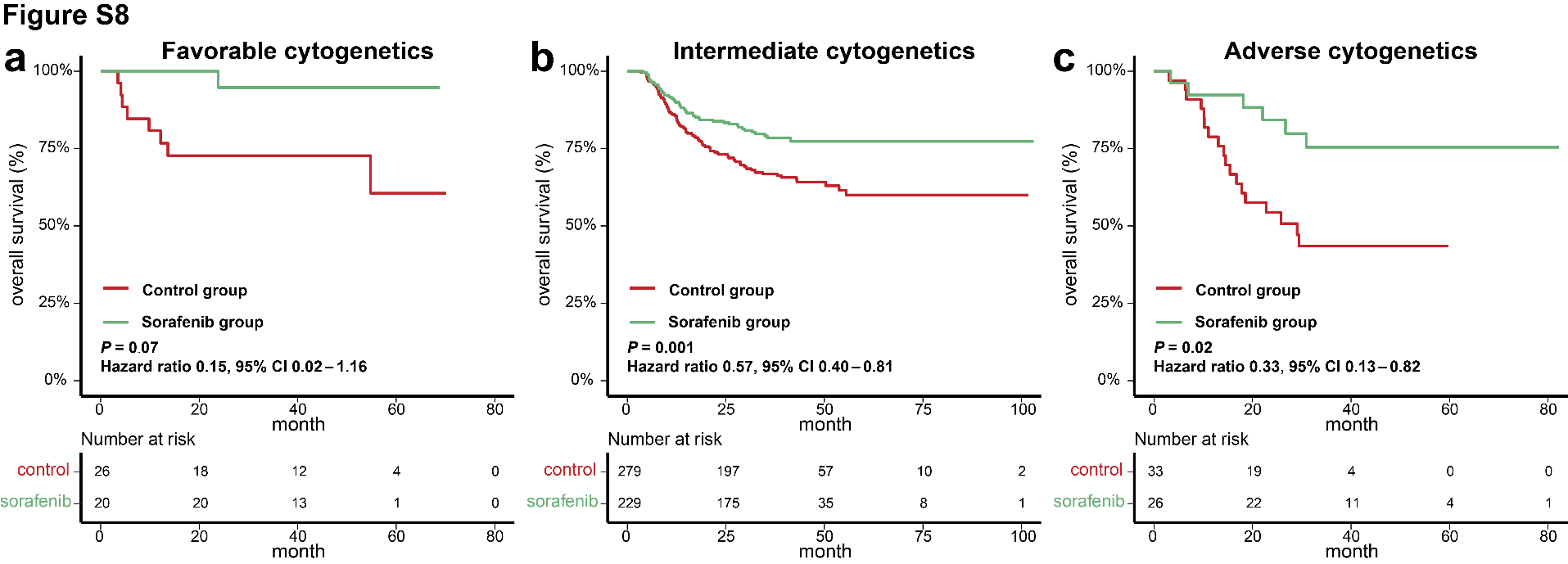


Figure. S8.

**Comparisons of OS among groups A-D (based on use of sorafenib pre- and post-transplantation) in patients with (a) favorable cytogenetics, (b) intermediate cytogenetics, and (c) adverse cytogenetics.**


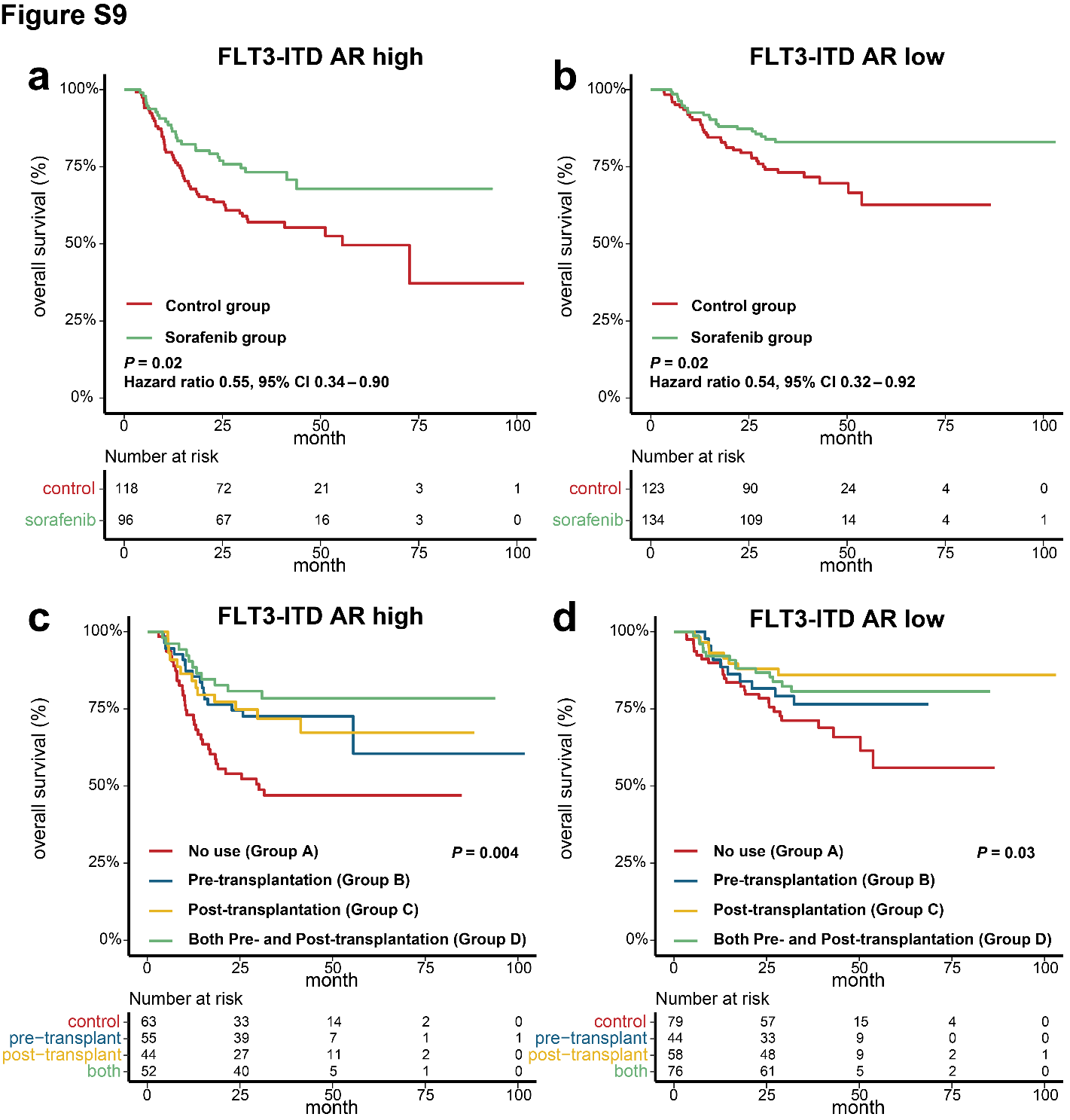


Figure. S9.

**Overall survival in FLT3-ITD AR subgroups.** Overall survival of the sorafenib and control groups in patients with (a) FLT3-ITD^high^ and (b) FLT3-ITD^low^. Overall survival among groups A-D in patients with (c) FLT3-ITD^high^ and (d) FLT3-ITD^low^.


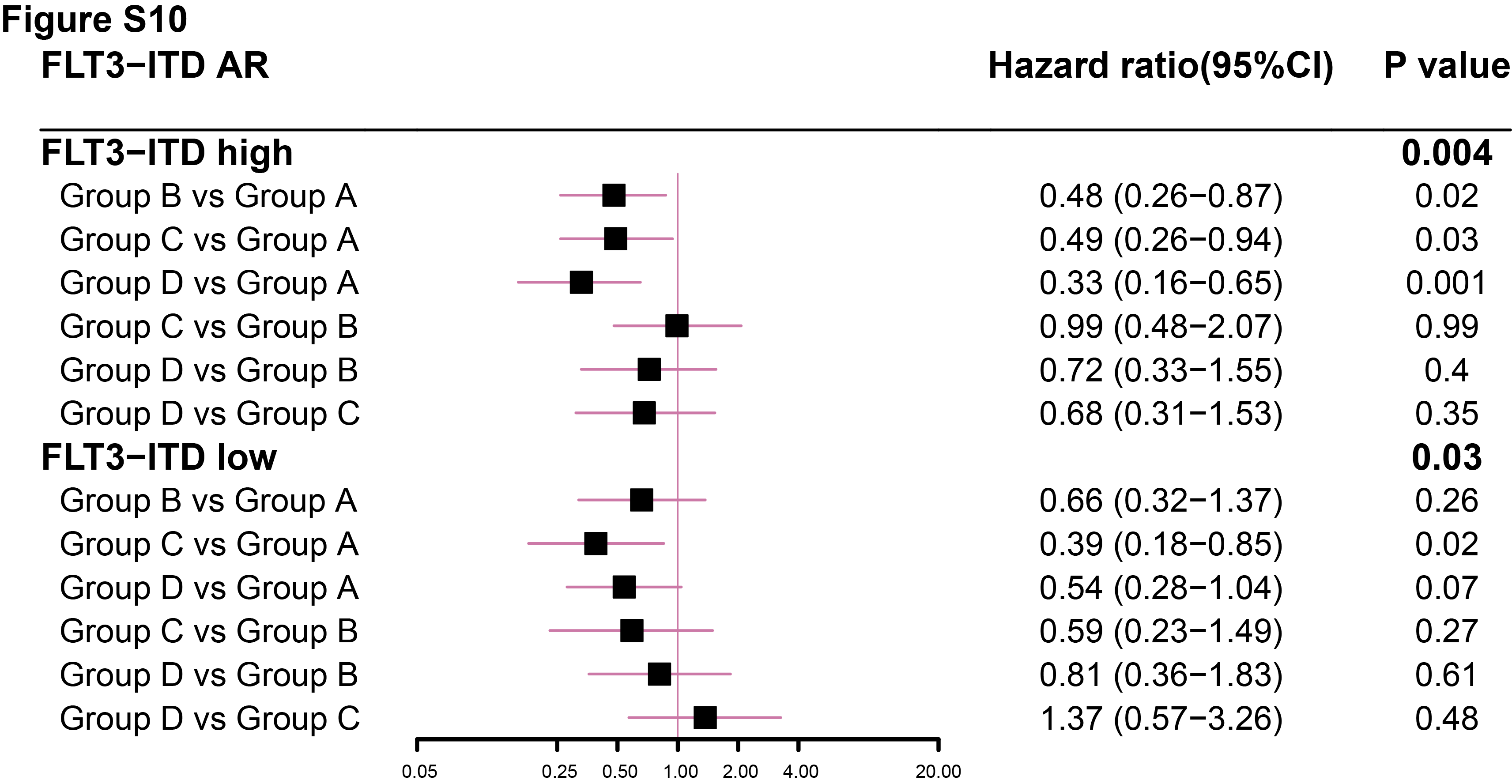


Figure. S10.

**Hazard ratios of OS in FLT3-ITD AR subgroups among groups A-D.**


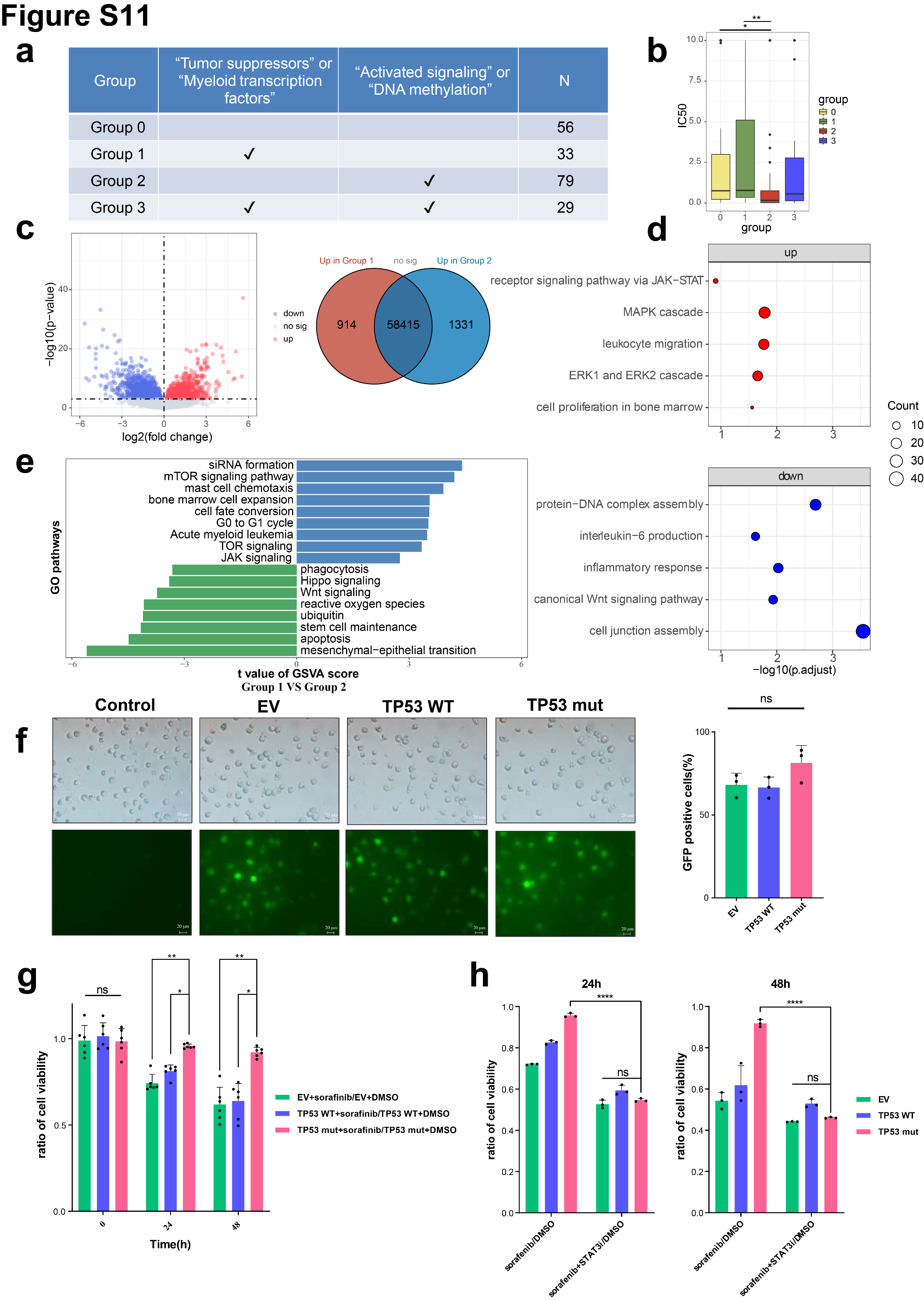


Figure. S11.

**Tumor suppressors and myeloid transcription factors mutations correlate with increased sorafenib resistance in FLT3-ITD AML.** (a) Grouping criteria of cases by concomitant mutations. (b) IC50 of the patients in the 4 groups to sorafenib. (c) Volcano plot for the differentially expressed genes in group 1 vs group 2. Venn plot shows the number of significantly differentially expressed genes in the two groups. (d) Comparison of the GSVA results on GO terms in group 1 vs group 2. (e) Results of GO enrichment analyses using significantly up- and down-regulated genes in group 1 vs group 2. (f) GFP florescence of MV411 cells electrotransfected with wild type TP53, TP53 mutant, and empty vehicle. (g) Cell viability of MV411 cells electrotransfected with wild type TP53, TP53 mutant and empty vehicle treated with sorafenib compared with DMSO at 0, 24, and 48 h after treatment. (h) Cell viability of wild type TP53-, TP53 mutant-, and empty vehicle-transfected MV411 cell lines treated with sorafenib, with or without STAT3 inhibitors, compared with DMSO at 24 and 48 h after treatment. *, P < 0.05; **, P < 0.01; ***, P < 0.001; ****, P < 0.0001.

**
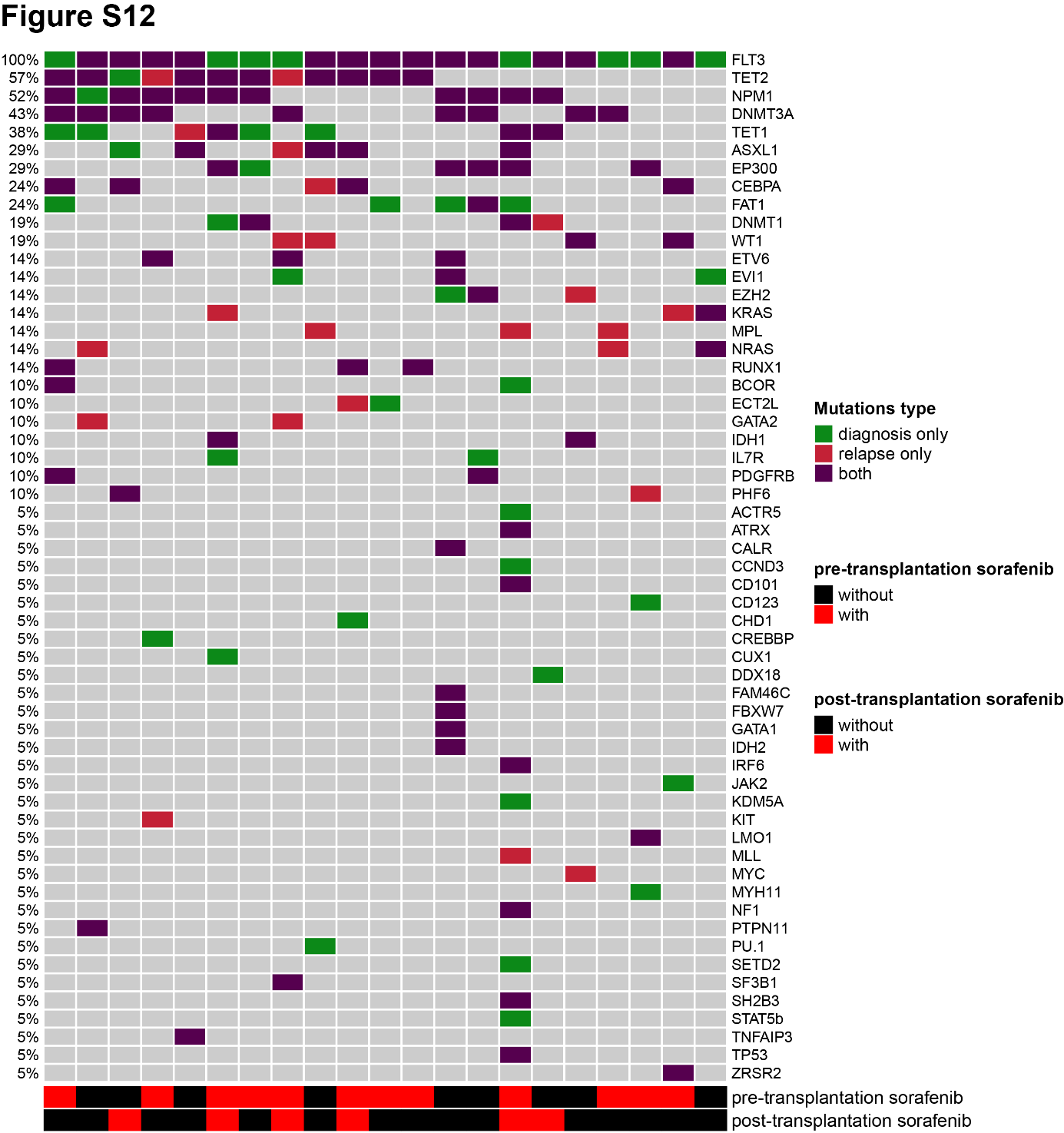
**

Figure. S12.

**Genetic patterns at diagnosis and relapse of 21 patients with paired NGS data at diagnosis and relapse**


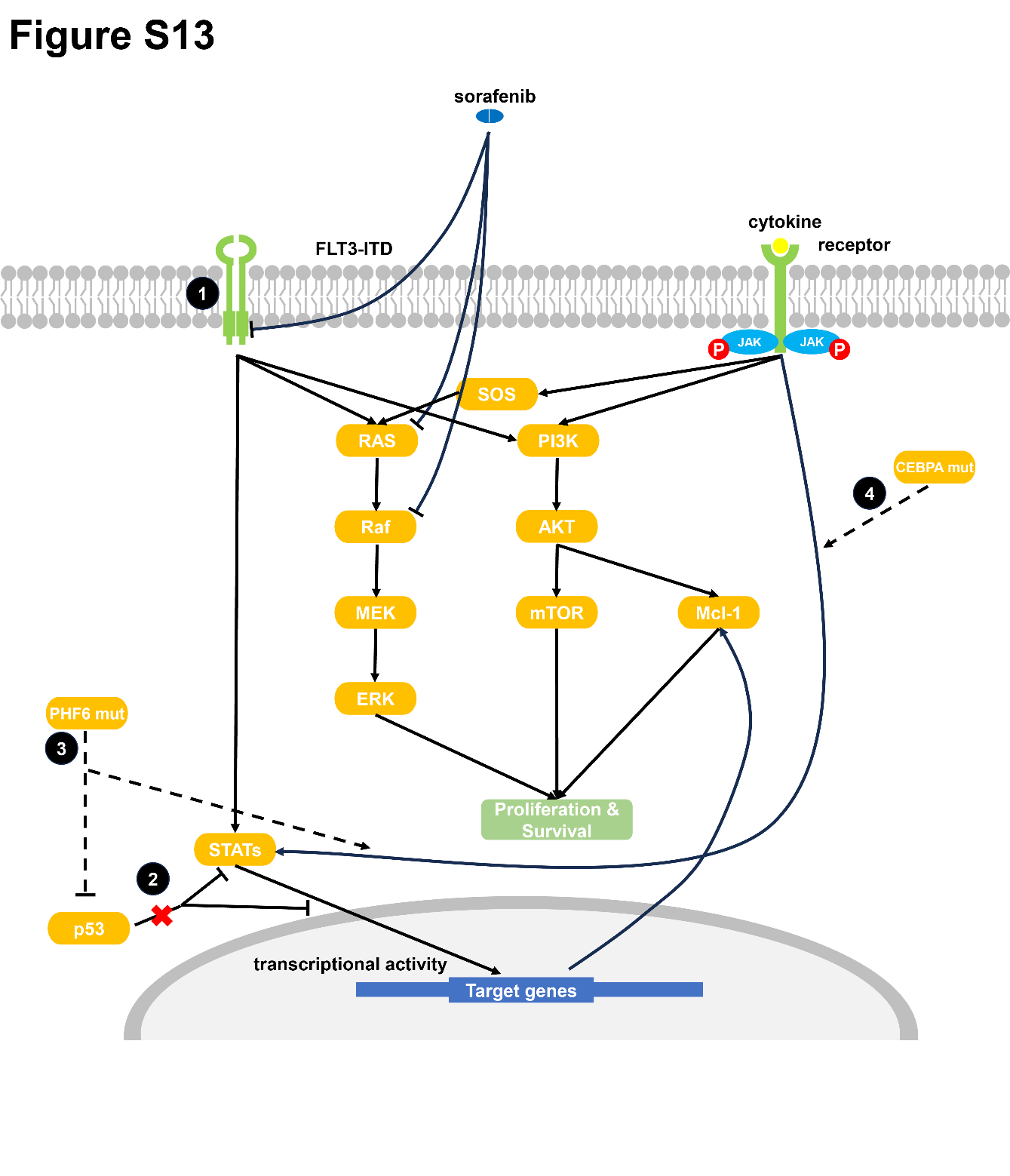


Figure. S13.

**Possible mechanisms of increased sorafenib resistance driven by tumor suppressors and myeloid transcription factors mutations.** ① FLT3-ITD mutation causes constitutive activation of FLT3 downstream signaling pathways including STATs, RAS/MAPK, and PI3K/AKT/mTOR, which promote the proliferation and survival of leukemia cells. ② Wild type p53 protein plays an inhibition role in both the phosphorylation and transcriptional activity of STAT proteins, especially STAT3 and STAT5, which can be lost through TP53 mutation. ③ PHF6 loss-of-function mutation or knock-out causes upregulation of the JAK-STAT signaling pathway and improves the ubiquitination-mediated degradation of p53 protein in T-cell acute lymphoblastic leukemia. ④AML with mutated CEBPA associates with a phenotype of JAK-STAT signaling activation and responses to JAK inhibitors.

Table S1.

**Clinical and Treatment Characteristics of the Prospective and Retrospective Cohort.**

| **Characteristics** | **Prospective cohort (n=441)** | **Retrospective cohort (n=172)** | **P** |
| --- | --- | --- | --- |
| **Sex, male/female (n%)** | 225(51%)/216(49%) | 94(55%)/78(45%) | 0.65 |
| **Age, median (IQR), years** | 36(27-46) | 35(27-45) | 0.29 |
| **WBC count at diagnosis, median (IQR)** | 53.1(15.2-121.8) | 51.3(13.6-110.0) | 0.29 |
| **Cycles of chemotherapy pre-transplant, median (IQR)** | 3(3-4) | 4(3-5) | < 0.001 |
| **Initial induction regimens** |  | | 0.82 |
| **Anthracyclines plus cytarabine** | 405(92%) | 157(91%) |  |
| **Others** | 36(8%) | 15(9%) |  |
| **Cytogenetics risk stratification (n%)** |  | | 0.13 |
| **Favorable** | 39(9%) | 7(4%) |  |
| **Intermediate** | 360(82%) | 148(86%) |  |
| **Adverse** | 42(9%) | 17(10%) |  |
| **2017 ELN risk stratification (n%)** |  | | 0.001 |
| **Favorable** | 132(30%) | 29(17%) |  |
| **Intermediate** | 101(23%) | 57(33%) |  |
| **Adverse** | 141(32%) | 46(27%) |  |
| **Unknown** | 67(15%) | 40(23%) |  |
| **Disease status at transplant (n%)** |  | | 0.56 |
| **CRc** | 381(87%) | 144(84%) |  |
| **PR** | 19(4%) | 7(4%) |  |
| **NR** | 41(9%) | 21(12%) |  |
| **Sorafenib pre-transplant (n%)** |  | | 0.14 |
| **Use** | 209(47%) | 70(41%) |  |
| **No use** | 232(53%) | 102(59%) |  |
| **Transplant modality (n%)** |  | | 0.52 |
| **MSD** | 174(40%) | 77(45%) |  |
| **MUD** | 27(6%) | 15(9%) |  |
| **HID** | 240(54%) | 80(46%) |  |

WBC, white blood cell; CRc, composite complete remission; PR, partial remission; NR, non-remission; MSD, HLA-matched sibling donor; MUD, HLA-matched unrelated donor; HID, HLA-haploidentical donor.

Table S2.

**Details of the 5 largest genetic pattern subgroups in different ELN risk groups.**

| **Favorable** | **Number of patients** | **Description** |
| --- | --- | --- |
| **Favorable-CBFs** | 46 | Favorable risk; with CBFβ-MYH11 or RUNX1-RUNX1T1 |
| **Favorable-CEBPA biallelic** | 30 | Favorable risk; with CEBPA biallelic mutation |
| **Favorable-DNMT3A** | 34 | Favorable risk; with DNMT3A mutation |
| **Favorable-NPM1** | 91 | Favorable risk; with NPM1 mutation |
| **Favorable-TET2** | 29 | Favorable risk; with TET2 mutation |

| **Intermediate** | **Number of patients** | **Description** |
| --- | --- | --- |
| **Intermediate-DNMT3A** | 28 | Intermediate risk; with DNMT3A mutation |
| **Intermediate-FLT3-ITD^low^ NPM1^WT^** | 52 | Intermediate risk; with FLT3-ITD^low^ and wild type NPM1 |
| **Intermediate-FLT3-ITD^high^ NPM1^mut^** | 57 | Intermediate risk; with FLT3-ITD^high^ and NPM1 mutation |
| **Intermediate-MLL** | 13 | Intermediate risk; with MLL mutation |
| **Intermediate-TET2** | 17 | Intermediate risk; with TET2 mutation |

| **Adverse** | **Number of patients** | **Description** |
| --- | --- | --- |
| **Adverse-ASXL1** | 26 | Adverse risk; with ASXL1 mutation |
| **Adverse-Cytogenetics** | 58 | Adverse risk; with adverse cytogenetics, including monosomal karyotype, complex karyotype, DEK-NUP214, MLL rearrangements, del(5q), minus(7), and GATA2, MECOM |
| **Adverse-FLT3-ITD^high^ Only** | 44 | Adverse risk; with FLT3-ITD^high^ and NPM1 mutation |
| **Adverse-RUNX1** | 28 | Adverse risk; with MLL mutation |
| **Adverse-TET2** | 32 | Adverse risk; with TET2 mutation |

Table S3.

**Gene ontology groups.**

| **Activated signaling** | **DNA methylation** | **Transcription** | **Tumor suppressor** | **Myeloid transcription factors** | **Chromatin modifiers** |
| --- | --- | --- | --- | --- | --- |
| NRAS | DNMT3A | DDX18 | PHF6 | CEBPA | ASXL1 |
| PTPN11 | TET2 | DDX3X | TP53 | ETV6 | EZH2 |
| PDGFRA | IDH2 | BCL6 | WT1 | GATA2 | MLL |
| PDGFRB | TET1 | FAM46C |  | RUNX1 | MLL2 |
| KIT | IDH1 |  |  |  | MLL3 |
| KRAS | DNMT1 |  |  |  | MLL5 |
| JAK1 |  |  |  |  | BCOR |
| JAK2 |  |  |  |  | ACTR5 |
| JAK3 |  |  |  |  | IRF6 |
| BRAF |  |  |  |  | KDM5A |

Table S4.

**The 167-gene next generation sequencing panel.**

| ABCA12 | CBL | CYLD | FAT4 | JAK3 | MYC | PRPF40B | SRSF2 | UBA2 |
| --- | --- | --- | --- | --- | --- | --- | --- | --- |
| ABL1 | CCND1 | DDB1 | FBXW7 | KDM2B | MYD88 | PTEN | SSPO | WHSC1 |
| ABL2 | CCND3 | DDX18 | FGFR3 | KDM5A | MYH11 | PTPN11 | STAG1 | WT1 |
| ACTR5 | CD101 | DDX3X | FLT3 | KDM6A | NF1 | PU.1 | STAG2 | XBP1 |
| AKT1 | CD123 | DIS3 | GATA1 | KIT | NFKB2 | RAD21 | STAT3 | XPO1 |
| ALK | CD79b | DNM2 | GATA2 | KRAS | NOTCH1 | RB1 | STAT5a | ZEB2 |
| ANK3 | CDC27 | DNMT1 | GATA3 | LMO1 | NOTCH2 | RELN | STAT5b | ZRSR2 |
| ARID1A | CDK4 | DNMT3A | GNAS | LMO2 | NPM1 | RHOA | SUZ12 |  |
| ARID2 | CDKN2A | EBF1 | HMGA2 | MAP2K1 | NRAS | ROS1 | SYK |  |
| ASXL1 | CDKN2B | ECT2L | HMGB1 | MDM2 | NT5C2 | RUNX1 | TAL1 |  |
| ATM | CEBPA | EED | HRAS | MEF2B | NUP98 | RUNX2 | TCF3 |  |
| ATRX | CHD1 | EGFR | ID2 | MLL | PAX5 | SAMHD1 | TERC |  |
| BAFF | CRBN | EP300 | IDH1 | MLL2 | PDGFRA | SETBP1 | TET1 |  |
| BCL2 | CREBBP | EPHA7 | IDH2 | MLL3 | PDGFRB | SETD2 | TET2 |  |
| BCL6 | CRLF2 | ERG1 | IKZF | MLL5 | PHF6 | SF1 | TIM-3 |  |
| BCOR | CSF1R | ETV6 | IL7R | MMD2 | PI3KCA | SF3A1 | TLX3 |  |
| BIRC3 | CSF3R | EVI1 | IRF4 | MN1 | PICALM | SF3B1 | TNFAIP3 |  |
| BRAF | CUX1 | EZH2 | IRF6 | MPL | PKM2 | SH2B3 | TP53 |  |
| CALR | CXCL12 | FAM46C | JAK1 | MTAP | PRDM1 | SOCS1 | TRAF3 |  |
| CARD11 | CXCR4 | FAT1 | JAK2 | MUM1 | PRMT5 | SOX4 | U2AF1 |  |
